# Elucidating the Chemistry Behind Thiol-Clickable GelAGE Hydrogels for 3D Culture Applications

**DOI:** 10.3390/gels11110874

**Published:** 2025-11-01

**Authors:** Sara Swank, Peter VanNatta, Melanie Ecker

**Affiliations:** 1 Department of Biomedical Engineering, University of North Texas, Denton, TX 76203, USA; saraswank@my.unt.edu; 2 Department of Chemistry, University of North Texas, Denton, TX 76203, USA; peter.vannatta@unt.edu

**Keywords:** thiol-click chemistry, ECM hydrogel, UV photopolymerization, degree of functionalization, NMR spectroscopy, tunable stiffness hydrogels, 3D cell culture, tissue engineering, thiol-ene hydrogels

## Abstract

Although covalently crosslinked gelatin hydrogels have been investigated for use in 3D cell culture due to inherent bioactivity and proliferation within the denatured collagen precursor, the stability of the matrix, and relatively inexpensive synthesis, current systems lack precise control over mechanical properties, including homogeneity, stiffness, and efficient diffusion of nutrients to embedded cells. Difficulties in modifying gel matrix composition and functionalization have limited the use of covalently crosslinked gelatin hydrogels as a three-dimensional (3D) cell culture medium, lacking the ability to tailor the microenvironment for specific cell types. In addition, the currently utilized chain-growth photopolymerization mechanism for crosslinking hydrogels has a potential for side reactions between the matrix backbone and components of the cell surface, requires a high concentration of radicals for initiation, and only cures with long irradiation times, which could lead to cytotoxicity. To overcome these limitations, a superfast curing reaction mechanism, in which a thiol monomer reacts efficiently with non-homopolymerizable alkenes, is suggested. This mechanism reliably produces a well-defined matrix that does not require a high radical concentration for photoinitiation. Mechanical customization of the hydrogel is largely achievable through variation in degree of functionalization of the gelatin backbone, dependent on reaction conditions such as pH, allyl concentration, and time. This work provides a mechanistic framework for GelAGE hydrogel fabrication by elucidating the molecular mechanism of gelatin functionalization with AGE and the thiol-ene crosslinking reactions controlling network stiffness. These insights provide the foundation for engineering hydrogels that mimic the viscoelastic and structural characteristics of cartilage, enabling advanced in vitro models for osteoarthritis research.

## 1. Introduction

Osteoarthritis (OA), a degenerative disease causing joint pain and stiffness, affects a large portion of the population, increasing in global prevalence by 113% since 1990 due to the rise in obesity, injury rates, and average life expectancy. As movement becomes labored, patients with osteoarthritis often restrict participation in meaningful activities, leading to a decline in both mental and physical health [1]. While symptoms can be managed with non-steroidal anti-inflammatory drugs, rehabilitation, and surgical intervention, the mechanisms behind OA initiation and progression are not well understood, and there are no currently available treatments effective in reducing or reversing disease progression [2]. Direct characterization of chondrocyte behavior within a biologically relevant in vitro platform will aid in our understanding of OA pathology and drive future therapeutic development.

**Figure 1:**
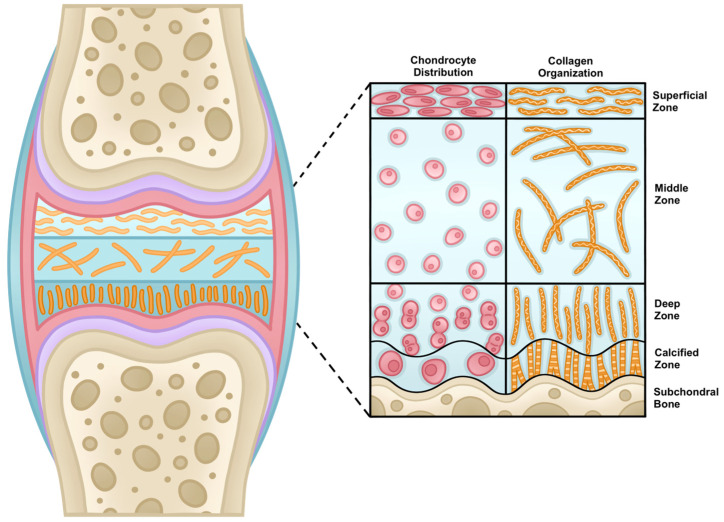
Layered ECM structure of articular cartilage, exhibiting higher stiffness near the bone interface.

Articular (hyaline) cartilage is an avascular, aneural connective tissue composed of extracellular matrix (ECM) maintained by a sparse distribution of chondrocytes localized in lacunae. As seen in Figure 1, it has a layered structure, exhibiting higher stiffness as type II and type X collagen density increases near the bone interface [3,4].

**Figure 2:**
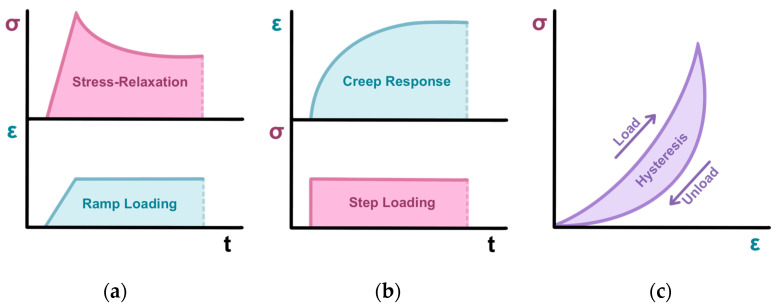
Characteristic biphasic viscoelastic behavior of articular cartilage: (**a**) Stress–relaxation response to ramp loading; (**b**) Creep response to step loading; (**c**) Energy dissipated by cyclic compression of viscoelastic material.

Due to the high water content of orthopedic tissues, articular cartilage shows biphasic viscoelastic behavior under compressive loading, which acts to protect the chondrocytes and subchondral bone from biomechanical stress associated with daily movement [4]. When a force is applied, the interstitial fluid first creates a hydrostatic pressure gradient, followed by a volumetric change as it flows out through the ECM (solid phase), encountering high frictional resistance (drag) that is inversely proportional to the low permeability and small pore size of the tissue. Finally, the ECM macromolecular network itself exhibits a linear elastic stress–strain response. The biphasic viscoelastic behavior of cartilage is visualized in Figure 2. Under increasing displacement (ramp, Figure 2a), cartilage exhibits a linear rise in force. Once desired compression is achieved and maintained, cartilage exhibits stress–relaxation response to equilibrium force. When force is suddenly applied (stepwise, Figure 2b), cartilage exhibits creep, a transient increase in deformation until equilibrium is reached [5]. In response to biomechanical feedback, chondrocytes regulate ECM production to maintain homeostasis. In healthy articular cartilage, cyclic compressive loading promotes chondroprotection and chondroinduction through upregulation of anabolic factors. In contrast, cartilage degeneration is a vicious cycle, initiated by a homeostatic disruption that causes a decrease in ECM production by chondrocytes, subsequently limiting their ability to cope with repetitive biomechanical stress [6,7].

Due to high swelling capacity of the three-dimensional (3D) polymeric network, hydrogels exhibit similar biphasic mechanical behavior when compressed, tunable through changes in pore size, crosslinking density, and hydrophobicity of the matrix. Gelatin, derived from acidic (type A) or alkaline (type B) hydrolysis of collagenous tissues, serves as a natural, biocompatible polymer for hydrogel fabrication [8]. In vivo, polypeptide chains are held together through hydrogen bonding of adjacent amino acids, most notably the bioactive arginine–glycine–aspartic acid (RGD) motif, which facilitates cell adhesion and growth. The utility of gelatin in practical applications is limited by low stability and poor mechanical strength at physiological temperatures due to the reliance on physical intermolecular interactions [9,10]. While some research has focused on stabilizing the reversible matrix through interpenetrated polymeric networks or addition of nanocomposite materials to preserve self-healing behavior, degradation under physiological conditions remains a challenge for long-term investigations [11,12].

Covalently crosslinked gelatin hydrogels (ex: gelatin methacrylate, GelMA) have been investigated for use in 3D cell culture due to inherent bioactivity and proliferation within the denatured collagen precursor, the stability of the matrix, and relatively inexpensive synthesis, but current systems lack precise control over mechanical properties, including homogeneity, stiffness, and efficient diffusion of nutrients to embedded cells. Difficulties in modifying backbone functionalization have limited the use of GelMA hydrogels as a 3D cell culture medium, lacking the ability to precisely tailor the microenvironment for specific cell types as mechanical properties are largely influenced by precursor density due to the homopolymerization of the network without use of an additional chemical linker [13]. In addition, the currently utilized chain-growth photopolymerization mechanism for crosslinking GelMA hydrogels has a potential for side reactions between the matrix backbone and components of the cell surface, requires a high concentration of radicals for initiation, and only cures with long irradiation times, which could lead to cytotoxicity [14,15,16,17]. Reaction with ambient oxygen to form peroxyl radicals may lead to interruption or termination of polymerization, leading to incomplete crosslinking and poor shape fidelity in bioprinted constructs [13].

**Figure 3:**
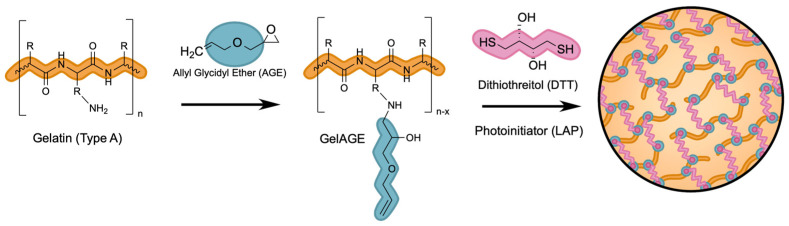
Functionalization of gelatin type A with AGE to form GelAGE hydrogel precursor, and subsequent UV crosslinking with DTT photoinitiated by LAP.

To overcome these limitations, an efficient, superfast curing reaction mechanism, utilizing thiol-ene click chemistry, has recently been advanced for the synthesis of gelatin-based hydrogels. This platform allows the use of a wide variety of thiol monomers (including dithiothreitol (DTT)) to react efficiently with non-homopolymerizable alkenes (allyl glycidyl ether (AGE)) that are coupled to the gelatin backbone, reliably producing a well-defined matrix that does not require a large radical concentration for photoinitiation [18,19,20]. GelAGE, the proposed thiol-clickable hydrogel system, is depicted in Figure 3 below, and a detailed description of the thiol-ene radical coupling mechanism is provided in supplemental documentation (Figure S1, Table S1). While this system has been explored for cell-related applications [20,21], it is not yet well understood how the reaction conditions influence the degree of substitution nor the exact location of the AGE introduction to the gelatin backbone. This study took a deep dive into the reaction mechanism to fully understand the chemistry behind the functionalization of gelatin type A with AGE to be able to precisely tune the system to achieve well-defined hydrogels that will enable us to create 3D cell cultures that replicate human cartilage in the future.

## 2. Results and Discussion

The ultimate goal of our research is to develop a highly tunable hydrogel system that utilizes gelatin for good cell adhesion combined with the highly efficient thiol-ene reaction to create materials that replicate cartilage. Before we can employ this technology, the gelatin backbone needs to be modified with either thiol or alkene moieties. For this research, we have focused on the latter through functionalization of gelatin type A with allyl glycidyl ether (AGE), which was previously described in the literature [20,21]. Mechanical customization of the hydrogel is largely achievable through variation in degree of functionalization of the gelatin backbone, dependent on reaction conditions such as pH, allyl concentration, and time, as well as the length and branching of the crosslinker [22].

### 2.1. Characterization of GelAGE Hydrogel Precursors

Variations in reaction conditions used in the synthesis of GelAGE hydrogel precursors for investigation are summarized below in Table 1. The GelAGE sample name corresponds to the reaction time (in hours), the concentration of NaOH (L = low, M = medium, H = high), and concentration of AGE functional groups (L = low, M = medium, H = high). For example, GelAGE-8LL corresponds to a low concentration of both NaOH (0.4 mmol per g gelatin) and AGE (2.4 mmol per g gelatin), reacted for 8 h at 65 °C (under constant magnetic stirring at 500 rpm).

#### 2.1.1. Nuclear Magnetic Resonance (NMR) Spectroscopy

**Figure 4:**
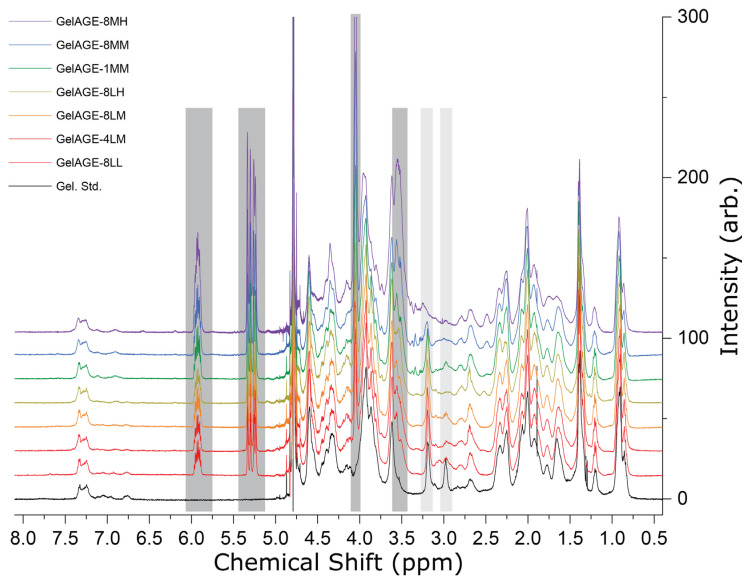
Stacked ^1^H NMR (D_2_O, 298 K) spectra of GelAGE samples. Spectra are normalized to the isolated aromatic region (7.05–7.35 ppm). Spectra are arranged to show the progression of functionalization, with highlighted regions in dark grey indicating the emergence of new spectral features due to the incorporation of AGE. Attenuation of diagnostic peaks of residue protons (2.98 ppm, LYS-ε and 3.19 ppm, ARG-δ), verified by 1D-TOCSY, are highlighted in light grey, reflecting successful functionalization at the associated residues dependent upon respective reaction conditions.

NMR spectroscopy was utilized to confirm successful functionalization of the gelatin with AGE, determine relative degree of functionalization (DOF), and unambiguously identify residue(s) functionalized. ^1^H and ^13^C spectra for all reaction conditions unambiguously show diagnostic features consistent with successful incorporation of the AGE functional group depicted in Figure 4. Specifically, a quartet of triplets attributable to the vinylic proton is observed at 5.86–5.98 ppm (Figure S2); a doublet of doublets attributable to the geminal protons is observed at 5.22–5.35 ppm; a doublet attributable to the alkene/ether-adjacent methylene protons is observed at 4.06 ppm (Figure S3); a broad feature attributable to the alcohol-bearing alkyl proton is observed at 3.95 ppm; and a broad feature attributable to the remaining ether-adjacent and amine-adjacent alkyl protons is observed at 3.45–3.60 ppm. ^13^C spectra were of marginal quality despite accumulation of 32,768 transients; this can be tentatively attributed to aggregation affects drastically shortening the effective transverse relaxation time (T2 *) as evidenced by free induction decay (FID) signal loss within 20 ms (Figure S4). Nonetheless, the GelAGE-4LM ^13^C NMR spectrum (Figure S5) showed signals at 134.2 ppm (vinylic -CH), 118.80 ppm (geminal -CH_2_), and 72.6 ppm (ether/alkane-adjacent -C). ^13^C-NMR of the GelAGE-8MM sample revealed two additional resonances, near the geminal proton feature at 118.80 ppm, consistent with three unique electronic environments. The two new features at 118.75 ppm and 118.70 ppm coalesce into a single feature at 118.70 ppm in the GelAGE-8MH spectrum (Figure S6).

**Figure 5:**
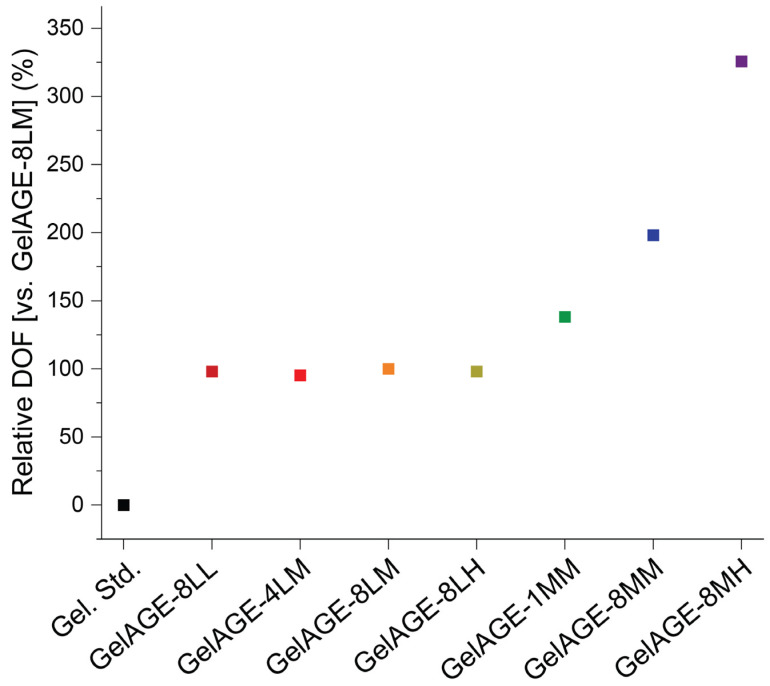
Degree of functionalization (DOF) relative to GelAGE-8LM, determined via integration of vinylic proton resonance region (5.72–5.86 ppm) of normalized ^1^H NMR (D_2_O, 298K) GelAGE sample spectra.

Due to aggregation effects observed in ^13^C spectra, and concern regarding the effect of aggregation on ^1^H-NMR quantitation of non-equivalent resonances, absolute quantification of DOF was determined to be outside the scope of this study. Nonetheless, relative DOF for each condition was calculated using the integration of the vinylic proton resonance relative to GelAGE-8LM and is presented in Figure 5. Functionalization of GelAGE samples produced via reaction conditions 8LL, 4LM, 8LM, and 8LH was found to be consistent within 5% deviation, indicating neither increased concentration of AGE nor increased time (4 h vs. 8 h) affords greater DOF with a 0.4 mmol NaOH per g gelatin loading concentration. GelAGE-1MM and GelAGE-8MM were found to be 38% and 98% more functionalized than GelAGE-8LM, respectively, indicating increased DOF as a function of reaction time (1 h vs. 8 h). GelAGE-8MH was found to be the most functionalized, 226% greater with respect to GelAGE-8LM, consistent with increasing DOF with increasing concentration of AGE at a 2.0 mmol NaOH per g gelatin concentration.

Due to the divergent DOFs of 1MM, 8MM, and 8MH with respect to the other conditions, identification of specific residues functionalized was evaluated by NMR. Previous reports describing AGE functionalization of gelatin have loosely assigned AGE addition to occur at lysine, hydroxylysine, and/or histidine [20] but lack detailed characterization to validate such assignments. Inspection of the 1H-NMR spectra between 2.3 ppm and 3.4 ppm revealed an apparent triplet at 2.98 ppm which is attenuated in all GelAGE spectra, coincident with the appearance of a new feature at 2.80 ppm (Figure S7). Selective 1D-TOCSY of the 2.98 ppm peak revealed strong correlation with a peak at 1.66 ppm and weaker correlation with a feature at 1.43 ppm (Figure S8), exclusively consistent with assignment as LYS-ε protons and affording unambiguous assignment of three peaks associated with unfunctionalized lysine in the gelatin standard (Table S2). In the samples with enhanced DOF, a feature at 3.19 ppm in the gelatin standard spectrum is similarly attenuated (Figure S7): partially in the GelAGE-1MM and GelAGE-8MM spectra, and fully in GelAGE-8MH spectrum, coincident with emergence of a new feature at 2.48 ppm. Selective 1D-TOCSY of the 3.19 ppm feature revealed moderate correlation with a peak at 1.63 ppm and weak correlation with a peak at 1.86 ppm, consistent with assignment of the 3.19 ppm feature to ARG-δ protons (Figure S9, Table S2). Selective 1D-TOCSY of the 2.48 ppm feature in GelAGE-8MH revealed strong correlation with peaks at 4.52 and 2.05 ppm and weaker correlation with a peak at 4.35 ppm, consistent with assignment of the 2.48 ppm feature as functionalized arginine *f*ARG-*β* protons (Figure S10). Consequently, many ^1^H resonances associated with LYS and ARG were assigned for the unfunctionalized and the functionalized gelatins (Table S2).

Further supporting evidence of arginine functionalization in GelAGE-1MM, 8MM, and 8MH can be observed in the ^13^C-NMR data, where the standard and representative GelAGE-4LM spectra show the presence of a feature at 157.1 ppm (Figure S11), only assignable to arginine-ζ or tyrosine-ζ carbons [23]. Given the ~2-fold lower tyrosine content (%*w*/*w*) relative to arginine in porcine gelatins [9], its ζ-carbon resonance is likely obscured by noise. The 157.1 ppm feature persists in the GelAGE-4LM spectra but is attenuated in the GelAGE-8MM spectra and not observed in the GelAGE-8MH spectra, consistent with partial and complete functionalization of the arginine residues. From the three unique geminal proton bearing carbon signals observed in the ^13^C-NMR at ~118.75 ppm in the GelAGE-8MM spectra and observation of partially retained feature diagnostic of ARG-δ protons at 3.19 ppm in the ^1^H-NMR spectrum of GelAGE-8MM, we posit the GelAGE-8MM sample consists of non-, singly, and doubly functionalized arginine residues. The coalescence of the two additional geminal proton bearing carbon features in GelAGE-8MM, relative to the GelAGE-8LX spectra (Figure S6), into a single feature in the GelAGE-8MH sample is consistent with primarily doubly functionalized arginine residues in GelAGE-8MH. Similarly, multiple features are observed at ca. 69.1 and 71.2 ppm in the GelAGE-8MM spectra (Figure S12); both sets coalesce to two individual peaks in the GelAGE-8MH spectrum and can be best ascribed to *f*ARG-δ and *f*ARG-α carbons, respectively. In addition to arginine functionalization, spectral features are consistent with full functionalization of lysine residues in GelAGE-1MM, -8MM, and -8MH.

**Figure 6:**
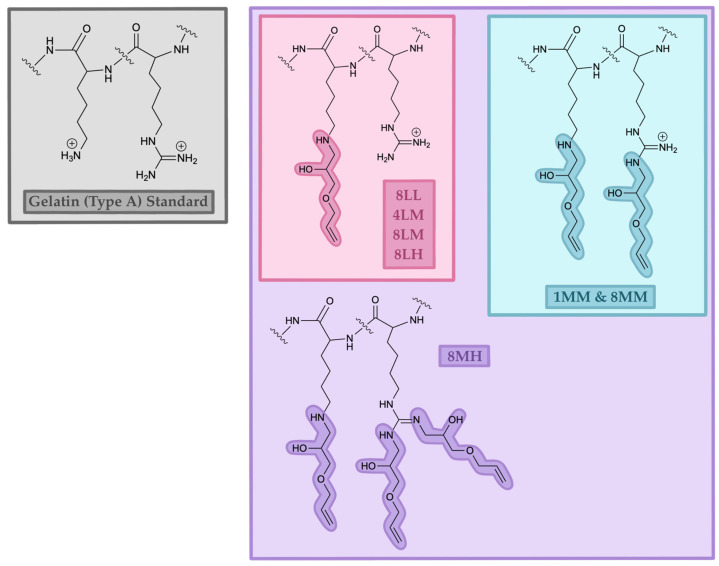
Proposed increasing functionalization of lysine and arginine residues in GelAGE samples. GelAGE-8LL, -4LM, -8LM, and -8LH samples display majority functionalization of only lysine residues (~7% total peptide residues). GelAGE-1MM and -8MM samples show full functionalization of lysine residues and partial functionalization of arginine residues. GelAGE-8MH exhibits majority functionalization of lysine residues and majority double-functionalization of arginine residues.

In summary, near-complete functionalization of lysine residues has been unambiguously observed in all GelAGE samples examined; under more alkaline conditions, additional partial (1MM, 8MM) or full (8MH) functionalization of arginine residues has been characterized, as illustrated in Figure 6. Notably, GelAGE-1MM/8MM reaction conditions show kinetic and stoichiometric control of arginine residue functionalization relative to GelAGE-8MH reaction conditions which result in near quantitative functionalization of all lysine and arginine residues. While the NMR data presented is short of quantitative, assuming ~7% (%*w*/*w*) lysine in porcine gelatin [9], we can be confident that the relative 100% DOF defined in this study can be estimated as approximately 7% total gelatin residue functionalization as all ^1^H-NMR spectra show near-complete attenuation of the diagnostic LYS-ε proton feature at 2.98 ppm. For other contexts, more mild conditions are necessary for sub-7% total DOF. Alternative hypotheses of functionalization at other residues under any reaction conditions, e.g., histidine or hydroxylysine, as have been suggested in previous reports [20], are not supported by NMR data presented in this study. Future work will be directed towards further control of functionalization and selective functionalization at other residues to facilitate increased stiffness of the crosslinked hydrogels.

#### 2.1.2. Gel Electrophoresis

**Figure 7:**
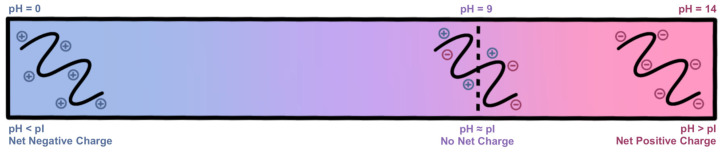
Isoelectric point of Type A (Porcine) gelatin, and the effects of pH on protein net charge.

Increasing the pH of the reaction to ≈9 (using 1 M NaOH), within the isoelectric region (pH ≈ 7–9) of type A gelatin (porcine), as illustrated in Figure 7, causes the protein chains to readily accept allyl functional groups [24]. Degree of functionalization is then dependent on available concentration of AGE, reaction time, and saturation limit. At higher concentrations of NaOH (pH > 10), however, concentration of negative charge along the gelatin backbone will cause the protein chains to repel one another, inhibiting the formation of tight *α*-helices, reducing the formation of non-covalent interactions, and producing a soft, easily compressible hydrogel. At pH ≈ 11 to 12, the backbone of gelatin may be compromised, resulting in peptide fragmentation [24].

**Figure 8:**
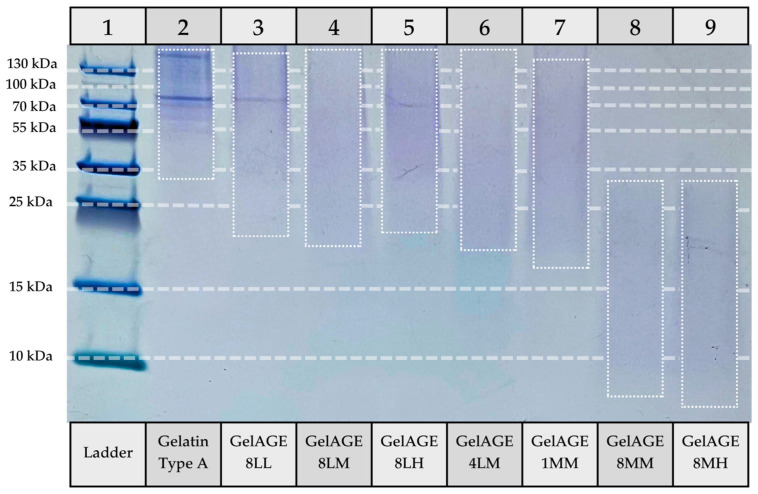
Approximate size distribution of Gelatin (Type A) standard and GelAGE hydrogel precursors in comparison to known ladder marker (PageRuler Plus, Invitrogen, Thermo Disher Scientific), obtained via SDS-PAGE (200 V, 30 min).

A qualitative estimate of molecular weight distribution was obtained via SDS-PAGE analysis of gelatin (type A) standard and GelAGE hydrogel precursors, shown below in Figure 8.

Specifically, while milder pH conditions (≈9 in GelAGE-8LL, -8LM, -8LH, and -4LM) show similar molecular weight distribution to unreacted gelatin, degradation of the gelatin backbone can be seen as the pH is increased (≈11). GelAGE-1MM shows the least primary structure disruption of the high-pH conditions, likely due to the relatively short reaction time. GelAGE-8MM and -8MH both appear as a faint smear, indicating a wide range of molecular weight fragments, with fragments less than 10 kDa present.

Coomassie Colloidal Blue binds to proteins via hydrophobic interactions with phenolic amino acids (tryptophan and phenylalanine) and electrostatic interactions with basic amino acids (arginine, lysine, and histidine) [25]. Although staining occurs via multiple binding domains, GelAGE-8MM and -8MH may show less affinity for the dye, thus lightening the band.

### 2.2. Characterization of GelAGE Hydrogels

The general mechanism of photoinitiated sulfur-ene radical coupling is well established [26], having first been reported by Kharasch, May, and Mayo in 1938 [27], and the mechanism remains accepted today [18]. A reasonable mechanism specific for the crosscoupling of GelAGE chains with DTT is presented in Figure S1. The coupling reaction is initiated by photolysis of the LAP anion with ~405 nm light generating a 2,4,6-trimethylbenzoyl radical and a phenylphosphonate radical anion [28,29]. Initiation of the thiol-ene reaction begins via homolytic cleavage of the thiol S-H bond. The products of hydrogen atom abstraction for the two products are 2,4,6-trimethylbenzaldehyde and phenylphosphonate anion. In comparison with other photoinitiation systems, e.g., [Ru(bpy)_3_]^2+^/persulfate [30], this pair of radicals are mild and competent for hydrogen atom transfer (HAT) from S-H moieties while presenting low cytotoxicity; though Nguyen and coworkers have reported cytotoxicity of LAP for a lithium-sensitive cell line [31]. Additionally, previous studies from our lab have shown that concentrations of LAP up to 0.1% (*w*/*v*) are non-toxic to TC28a2 chondrocytes in combination with 2 min of UV irradiation, which are cells of interest for OA-related research [32].

To better understand the “mildness” of the photogenerated radicals, their bond dissociation enthalpies (BDEs) and aqueous bond dissociation free energies (BDFE_(aq)_) were calculated using an experimentally calibrated method (see Section S2 for computational details). Briefly, the DFT-derived BDFE_(aq)_ of 2,4,6-trimethylbenzaldehyde (89.3 kcal/mol) is consistent with thermodynamically favorable HAT from the DTT S-H bond (BDFE_(aq)_ = 86.2 kcal/mol) by the 2,4,6-trimethylbenzoyl radical, while being disfavorable for HAT from amino acid X-H bonds, with few exceptions. In contrast, the DFT-derived BDFE_(aq)_ of the phenylphosphonate anion (84.2 kcal/mol) is insufficient for appreciable reactivity within the crosslinking reaction trajectory. These results are wholly consistent with experimental results reported in the literature (see Section S2 for full discussion). This “mildness” is in contrast to other visible-light photoinitiator systems such as [Ru(bpy)]^2+^/persulfate where the generated sulfate radicals have oxidizing potentials in line with the hydroxyl radical and therefore are competent for an expansive array of detrimental side-reactions [33].

#### 2.2.1. Compressive Modulus

**Figure 9:**
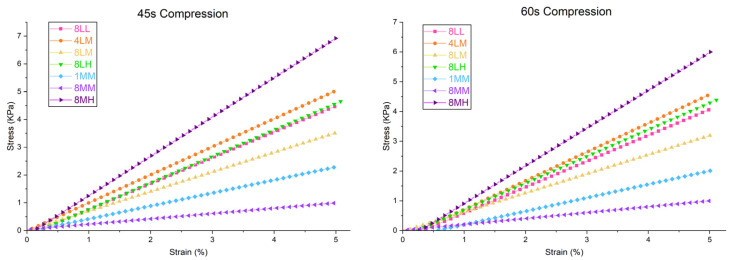
Comparison of averaged compressive moduli of saturated GelAGE hydrogels, collected under physiological conditions (aqueous, 37 °C) via MicroTester (CellScale).

The compressive moduli of crosslinked GelAGE hydrogel variants (Figure 9, Table 2) were determined as the slope of the linear region of the stress (KPa) vs. strain (%) curve, under ramp loading to 5% strain in physiological conditions (aqueous, 37 °C), as visualized in Video S1. Full analysis of the Young’s modulus of each GelAGE hydrogel variant during the 100 s cycles (45 s compression) and the 140 s cycles (60 s compression) can be found in Figures S13–S19.

In alignment with hydrogel precursor functionalization determined by NMR spectroscopy, GelAGE-8LL, -4LM, -8LM, and -8LH show similar stiffness under cyclic compressive loading. Although GelAGE-1MM shows a 38% increase in functionalization (compared to 8LM) due to pH-mediated addition of AGE on arginine residues, the compressive modulus was shown to decrease, likely due to fragmentation. This effect is magnified in GelAGE-8MM, which, despite a 98% increase in functionalization over -8LM, shows the lowest crosslinked stiffness. The degradation of the backbone is slightly countered in GelAGE-8MH, likely due to increased functionalization (226% increase over 8LM) compensating for the small fragment size.

#### 2.2.2. Degree of Swelling

**Figure 10:**
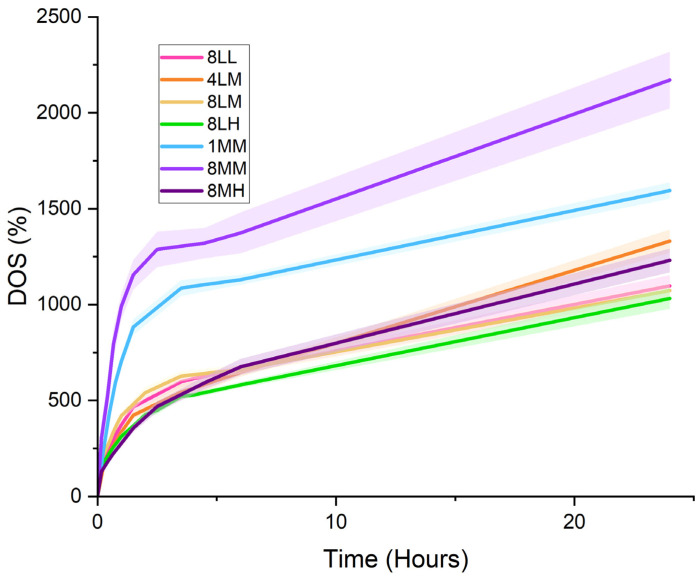
Degree of swelling (%) of lyophilized crosslinked GelAGE hydrogels from 0 to 24 h submerged in DI water at room temperature (20 °C).

The swelling behavior of crosslinked GelAGE hydrogel variants (Figure 10, Table 3) was characterized as the normalized ratio (%) of saturated weight (at time 0 h to 24 h) to the initial lyophilized weight, as visualized in Video S2.

As crosslinking density increases, the stiffness of the GelAGE hydrogel also increases. As a result, the relative water absorption by the matrix (DOS %) slows and decreases in total capacity due to decreased pore size. Following this principle, crosslinked GelAGE-8LL, -4LM, -8LM, and -8LH hydrogels show similar swelling behavior in accordance with their similar degree of functionalization determined via NMR spectroscopy, qualitative backbone degradation observed via SDS-PAGE, and compressive moduli. Hydrogels with lower stiffness (GelAGE-1MM and 8-MM) swelled quickly and retained a larger volume of DI water. Although GelAGE-8MH swelled at the slowest rate, it showed similar retention capacity to GelAGE-8LL, -4LM, -8LM, and -8LH.

#### 2.2.3. Scanning Electron Microscopy (SEM)

**Figure 11:**
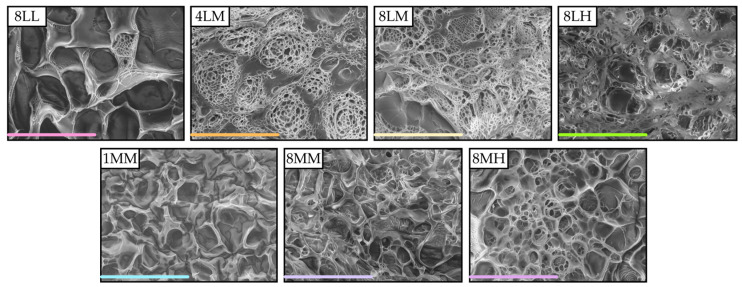
SEM imaging of lyophilized crosslinked GelAGE hydrogels at 250× magnification (Hitachi, Tokyo, Japan). Scale bars (located at the bottom left) represent 300 µm.

Morphological properties of the crosslinked GelAGE hydrogels were examined by SEM, as shown in Figure 11. Each matrix exhibited a relatively uniform porous structure, in which pore size was roughly associated with the observed mechanical and swelling behavior of each GelAGE variant. Most notably, hydrogels with lower compressive moduli and higher swelling ratios (GelAGE-1MM and 8-MM) showed a distinct lack of microporous substructures, which likely contribute to the ability to resist deformation in stiffer GelAGE hydrogel variants.

#### 2.2.4. Energy Dispersive X-Ray (EDX) Spectroscopy

**Figure 12:**
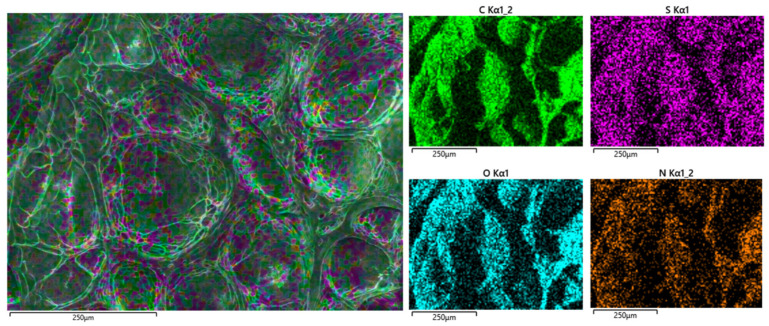
EDX mapping of lyophilized crosslinked GelAGE-4LM hydrogel (Oxford Instruments, Concord, MA, USA).

Local chemical composition of the lyophilized GelAGE hydrogel samples was characterized by EDX spectroscopy, as shown in Figure 12. All GelAGE hydrogel variants showed similar homogenous distribution of thiol crosslinker, as sulfur concentration increased from 0.23% (*w*/*w*) in gelatin (type A) standard to 1.12% (*w*/*w*) in GelAGE-4LM (Tables S3 and S4).

## 3. Conclusions

Through variations in pH, reaction time, and allyl group concentration during synthesis, the degree of functionalization of GelAGE hydrogel precursors can be modified. At the isoelectric point of gelatin (Type A, pH ≈ 9), functionalization is limited to lysine residues (≈7% *w*/*w* of porcine gelatin), independent of reaction time (4 h vs. 8 h) at the investigated AGE concentrations (2.4, 12, 24, and 48 mmol per g gelatin in 8LL, 8LM, 4LM, and 8LH variants, respectively). At higher pH (≈11), NMR spectroscopic analysis suggests that functionalization also occurs at arginine residues, first partially in GelAGE-1MM (38% increase in relative DOF compared to 8LM) due to a relatively short reaction time (1 h). GelAGE-8MM showed a 98% increase in functionalization relative to 8LM, suggesting near-complete single-substitution at arginine residues, which also comprise about 7% *w*/*w* of porcine gelatin [9]. Due to increased concentration of allyl moiety, GelAGE-8MH was able to achieve double-functionalization of arginine residues, exhibiting a 226% increase relative to 8LM. Although previous reports have suggested alternative hypotheses of functionalization at other residues, e.g., histidine or hydroxylysine [20], these are not supported by NMR data presented in this study.

Despite increased functionalization, GelAGE-1MM and -8MM hydrogel variants show lower crosslinking density with DTT, resulting in larger matrix pore size, lower mechanical stiffness under cyclic compressive loading, and increased swelling capacity. This is likely due to degradation of the gelatin backbone under alkaline conditions [24]. Although GelAGE-8MH also shows primary sequence disruption, it is partially overcome by the branched double-functionalization of arginine residues and shows increased crosslinking density compared to lysine-functionalized GelAGE hydrogel variants (8LL, 4LM, 8LM, and 8LH). As a result, GelAGE-8MH shows the highest compressive modulus (141.65 ± 3.02 KPa) and the slowest fluid diffusion of the investigated variants. Due to the heterogenous nature of articular cartilage, experimentally derived Young’s moduli vary significantly across studies, although a review of unconfined compression of healthy human cadaveric samples estimated 100 to 3490 kPa [34]. Following OA onset, decreased ECM production by overwhelmed chondrocytes leads to a further decrease in the reported dynamic modulus [6,7]. Although unseeded GelAGE hydrogels already fall within the acceptable stiffness range for physiological relevance, introduction of chondrocytes is expected to greatly increase compressive strength of engineered cartilage constructs, as porosity is replaced with approximately 10–15% (*v*/*v*) cells. High seeding density is associated with a similarly increased rate of scaffold replacement, effectively allowing the chondrocytes to customize their environment in response to biomechanical cues [35].

Future studies will be centered around the development of a biomimetic cartilage construct with embedded chondrocytes, in which layer-by-layer deposition of variable stiffness GelAGE hydrogels acts to recreate the hierarchical structure found in native articular cartilage. Although this study focused on characterization of GelAGE hydrogels crosslinked with DTT, variations in network density and stiffness are largely achievable through interchangeable thiol monomers, including tri- and tetra-branched structures. Orthogonal interpenetration of thiolated gelatin (GelSH) crosslinked with various diene monomers or direct crosslinking of GelAGE with GelSH precursors can further increase the range of mechanical properties achievable in engineered tissue constructs. Further investigation is needed to determine the chemical and temporal degradation profile of GelAGE hydrogels for induction of osteoarthritic conditions, verifying the physiological relevance and predictivity of the model as a platform for novel therapeutic development.

Although previous studies have shown good viability of chondrocytes embedded within homogenous stiffness GelAGE hydrogels, optimal culture conditions will be verified for the layered construct. Our lab has previously concluded that LAP photoinitiator is biocompatible at the proposed concentration with 2 min of UV irradiation [32], although kinetic evaluation of the reaction suggests that much lower concentrations of LAP may be employed utilizing ambient visible light for initiation to reduce potential cytotoxicity.

## 4. Materials and Methods

### 4.1. Synthesis of GelAGE Hydrogel Precursor

**Figure 13:**
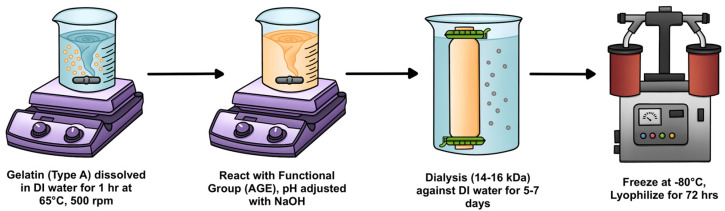
Schematic representing the synthesis of GelAGE hydrogel precursors.

To begin characterization of the thiol-clickable, gelatin-based hydrogel, GelAGE hydrogel precursors were synthesized according to an established protocol, with variations in reaction condition aimed at achieving different degrees of functionalization [20]. As illustrated in Figure 13, type A gelatin (10% *w*/*v*) from porcine skin (~300 g Bloom, Sigma-Aldrich, St. Louis, MO, USA) was fully dissolved in deionized (DI) water under magnetic stirring at 500 rpm for 1 h at 65 °C. Allyl glycidyl ether (AGE, Sigma-Aldrich) and 1M sodium hydroxide (NaOH, Sigma-Aldrich) were added in a drop-wise manner, and the reaction was carried out under constant stirring at 500 rpm for 1–8 h at 65 °C to induce allylation. The resulting solution was dialyzed through a cellulose membrane (molecular weight (MW) cutoff ≈ 12–14 kDa, Spectrum SpectraPor, Thermo Fisher Scientific, Waltham, MA, USA) against DI water at 37 °C for 5–7 days, changing the water every 12 h to ensure complete removal of unreacted impurities. The GelAGE precursor was stored at −80 °C overnight and then subsequently freeze-dried in a lyophilizer (Labconco, Kansas, MO, USA) for 72 h, generating a porous white foam that was stored at −80 °C until further use.

### 4.2. Characterization of GelAGE Hydrogel Precursors

#### 4.2.1. Nuclear Magnetic Resonance (NMR) Spectroscopy

To determine the relative functionalization, samples were first prepared in Norell XR-55-7 NMR tubes (Sigma-Aldrich, St. Louis, MO, USA) by dissolving gelatin type A (standard) and GelAGE hydrogel precursors in deuterium oxide (D_2_O) at a concentration of 10 mg/mL.

All NMR experiments were performed using a Varian 500 MHz spectrometer (Magnex Scientific Limited, Oxford, UK) running vnmrj v.3.2revC equipped with a 5 mm Dual Band Auto X probe (Varian, Palo Alto, CA, USA) at room temperature. Fourier transforms of compiled FID data were accomplished using MestreNova software (Version 16.0.0), using default auto-phase and baseline subtraction (3rd order Bernstein polynomials) functions. Chemical shifts are reported as parts per million (ppm) vs. tetramethylsilane (TMS), referenced against residual solvent (H_2_O: δ = 4.79 ppm). ^1^H spectra were collected using a standard single pulse sequence (“s2pul” with d2 = 0) with a spectral width of 8013 Hz, acquisition time of 2.0 s, relaxation delay of 1.0 s, and pulse angle of 45° and are presented as the summation of 64 transients. ^13^C spectra were collected using a standard single pulse sequence (“s2pul” with d2 = 0) with application of broadband ^1^H decoupling. Acquisition parameters included: spectral width of 31,250 Hz, acquisition time of 1.0 s, relaxation delay of 1.0 s, and pulse angle of 30° and are presented as a summation of 32,768 transients, with application of a 5 Hz exponential apodization function. Selective 1D-TOCSY experiments were conducted using the “TOCSY1D” pulse sequence with an MLEV-17 spin-lock, spectral width of 8013 Hz, acquisition time of 0.5 s, and relaxation delay of 1.5 s. Irradiation of the desired peak was performed using a selective 180° pulse applied to the selected band (35–36 Hz bandwidth) with an 80 ms mixing time; spectra are presented as a summation of 256 transients with application of a ≤3 Hz exponential apodization function.

#### 4.2.2. Gel Electrophoresis

To determine the approximate molecular weight of GelAGE hydrogel precursors, electrophoresis was conducted using a NuPAGE 4–12% Bis-Tris Gel (Invitrogen, Thermo Fisher Scientific, Waltham, MA, USA) according to previously established protocols [36].

Briefly, the polyacrylamide gel was affixed to an electrophoresis system and submerged in sodium dodecyl sulfate (SDS) running buffer. Gelatin (type A) standard and GelAGE precursor samples were dissolved in DI water at a concentration of 5 µg per 15 µL and mixed with loading buffer in a 3:1 ratio. The ladder marker (~10–250 kDa, PageRuler Plus Prestained Standard, Invitrogen, Thermo Fisher Scientific) and the resultant sample solutions (20 µL each) were loaded into each well, and 200 V (500 mA, 120 W) was applied for 30 min to ensure proper resolution. Once complete, the polyacrylamide gel was stained using a Coomassie Colloidal Blue staining kit (Invitrogen, Thermo Fisher Scientific). The gel was destained over a period of 3 days in DI water and subsequently photographed for analysis.

### 4.3. Fabrication and Characterization of GelAGE Hydrogels

#### 4.3.1. UV Crosslinking

**Figure 14:**
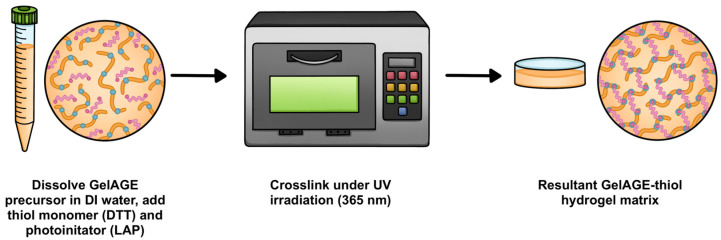
Schematic representing the synthesis of UV crosslinking of GelAGE hydrogels with thiol monomer (DTT) and photoinitiator (LAP).

After characterization of the hydrogel precursors, photopolymerization was performed by first dissolving GelAGE (10% *w*/*v*) in 1× phosphate-buffered saline (PBS) with agitation via a Vortex Mixer (ONiLab, Walnut, CA, USA) or planetary SpeedMixer (Hauschild, Farmington Hills, MI, USA). Thiol monomer (dithiothreitol, DTT; Sigma-Aldrich) was added in slight stoichiometric excess (≤6% w.r.t. 1 DTT: 2 AGE molar ratio), with each solution receiving an equal amount (0.2% *w*/*v*) of photoinitiator (lithium phenyl-2,4,6-trimethylbenzoylphosphinate, LAP; Sigma-Aldrich). GelAGE hydrogels were then fabricated by dispensing 3 mL of each reaction solution into a 35 mm diameter polystyrene microplate (Thermo Fisher Scientific) via micropipette (ONiLab). The samples were cured for 2 min with 365 nm irradiation in a UVP crosslinker (CL-3000L, Analytik Jena, Jena, Germany). This process is summarized in Figure 14 below. Crosslinking was confirmed via inversion of the microplate, and the GelAGE hydrogel was considered fully cured if no free-flowing liquid was observed following UV irradiation. Samples for mechanical testing were removed from the bulk GelAGE hydrogels using a 4 mm diameter biopsy punch (MedBlades, Addison, IL, USA). Samples for swelling testing and SEM imaging were removed from the bulk GelAGE hydrogels using an 8 mm diameter biopsy punch (MedBlades), stored at −80 °C overnight, and then subsequently freeze-dried in a lyophilizer (Labconco) for 24 h.

#### 4.3.2. Compressive Modulus

To determine the stiffness of fully saturated GelAGE hydrogels, mechanical testing was performed under physiological conditions (aqueous, 37 °C) using a MicroTester (CellScale, Waterloo, ON, Canada). A tungsten microbeam (1.02 mm diameter) with an affixed platen (5 × 5 mm^2^) was equipped to the vertical actuator, and relative deflection was monitored during compression via real-time camera tracking. Two sets of experimental data were collected in triplicate for each sample, reaching a final strain of 5% via ramp increase in a 100 s cycle (45 s compression, 5 s hold, 45 s relaxation, and 5 s rest) and a 140 s cycle (60 s compression, 10 s hold, 60 s relaxation, and 10 s rest). The compressive modulus was determined as the slope of the linear region of the stress–strain curve.

#### 4.3.3. Degree of Swelling

The degree of swelling (DOS %) of the GelAGE hydrogels, and therefore the tendency to exhibit biphasic viscoelastic behavior mimetic of native articular cartilage, was monitored by rehydrating lyophilized samples in room temperature (20 °C) DI water and assessed via the following equation [37]:

(1) $$\mathrm{DOS}\;\%\;=\;[\frac{{(W}_{s}\;-\;W_{d})}{W_{d}}]\;\times \;100\%$$

where W_s_ represents the weight of saturated (rehydrated) hydrogel and W_d_ represents the initial weight of the lyophilized (freeze-dried) hydrogel. Weight measurements were taken every 10 min for the first hour, every 30 min for hours 2–6, and finally at 24 h using a B-2 Series Analytical and Precision Balance with Draft Shield (VWR International, Avantor, Radnor, PA, USA).

#### 4.3.4. Scanning Electron Microscopy (SEM)

The morphological properties of the lyophilized GelAGE hydrogel samples were characterized by scanning electron microscopy (SEM) utilizing a TM3030Plus (Hitachi, Tokyo, Japan) with an operating voltage of 15 kV.

#### 4.3.5. Energy Dispersive X-Ray (EDX) Spectroscopy

The local chemical composition of the lyophilized GelAGE hydrogel samples was characterized by energy dispersive X-ray (EDX) spectroscopy utilizing the Xplore Detector and AZtecOne (Version 3.3) processing software (Oxford Instruments, Concord, MA, USA).

## Figures and Tables

**Table 1:** GelAGE synthesis conditions to achieve variation in degree of functionalization.

GelAGE Variant	NaOH Concentration (mmol per g Gelatin)	AGE Concentration (mmol per g Gelatin)	pH
8LL 4LM	0.4 0.4	2.4 24	9 9
8LM	0.4	12	9
8LH 1MM	0.4 2.0	48 12	9 11
8MM	2.0	12	11
8MH	2.0	60	11

**Table 2:** Compressive moduli of crosslinked GelAGE hydrogels for 45 s compression.

GelAGE Variant	Compressive Modulus (KPa)
8LL 4LM	92.15 ± 2.71 100.93 ± 0.80
8LM	70.44 ± 1.14
8LH 1MM	95.31 ± 0.82 46.77 ± 0.57
8MM	19.16 ± 0.25
8MH	141.65 ± 3.02

**Table 3:** DOS (%) of crosslinked GelAGE hydrogels at 1h, 6 h, and 24 h.

GelAGE Variant	DOS (%) at 1 h	DOS (%) at 6 h	DOS (%) at 24 h
8LL 4LM	372.91 ± 19.91 335.24 ± 11.80	662.83 ± 19.57 646.52 ± 22.58	1097.91 ± 55.65 1331.07 ± 60.30
8LM	419.91 ± 20.51	661.86 ± 24.23	1073.40 ± 35.67
8LH 1MM	312.93 ± 18.54 703.8 ± 35.15	581.26 ± 14.13 1129.46 ± 25.70	1032.06 ± 53.68 1594.82 ± 42.71
8MM	990.12 ± 65.23	1374.01 ± 106.61	2170.94 ± 148.06
8MH	276.75 ± 18.63	675.39 ± 42.07	1230.58 ± 61.97

## Data Availability

The original contributions presented in this study are included in the article/Supplementary Material. Further inquiries can be directed to the corresponding author(s), and the raw data will be made available on request.

## Supplementary Materials

The following supporting information can be downloaded at: https://www.mdpi.com/article/10.3390/gels11110874/s1, Figure S1: Mechanism of thiol–radical coupling mechanism; Table S1: BDE and BDFE determined via computational methods; Figures S2–S12: Nuclear Magnetic Resonance (NMR) Spectroscopy; Table S2: Amino Acid Residue Chemical Shift Comparison Gelatin Standard vs. GelAGE; Video S1: Cyclic Compressive Testing using CellScale MicroTester (https://youtu.be/gp0m0c_BuFQ (accessed on 23 October 2025)); Figures S13–S19: GelAGE Young’s Moduli during 45 s and 60 s Compression; Video S2: Swelling of Lyophilized GelAGE Hydrogel (0 h to 6 h, https://youtu.be/w-gfS24I9BE (accessed on 23 October 2025)); Table S3: EDX map spectra of gelatin (type A) standard; Table S4: EDX map spectra of crosslinked GelAGE-4LM.
