# Supplementary material for: Elucidating the Chemistry Behind Thiol-Clickable GelAGE Hydrogels for 3D Culture Applications
Source: Gels. 2025 Nov 1;11(11):874. doi: 10.3390/gels11110874 (PMC12652731; doi:10.3390/gels11110874)
Supplement: Supplementary file 1 [file gels-11-00874-s001.zip › gels-3926349-supplementary.pdf]

gels

MDPI

SUPPLEMENTAL DATA

Sara Swank [1], Peter VanNatta [2] and Melanie Ecker [1,*]

[1] Department of Biomedical Engineering, University of North Texas, Denton, TX 76203, USA
[2] Department of Chemistry, University of North Texas, Denton, TX 76203, USA
* Correspondence: melanie.ecker@unt.edu

S.1. Detailed Description of the Thiol-ene Radical Coupling Mechanism

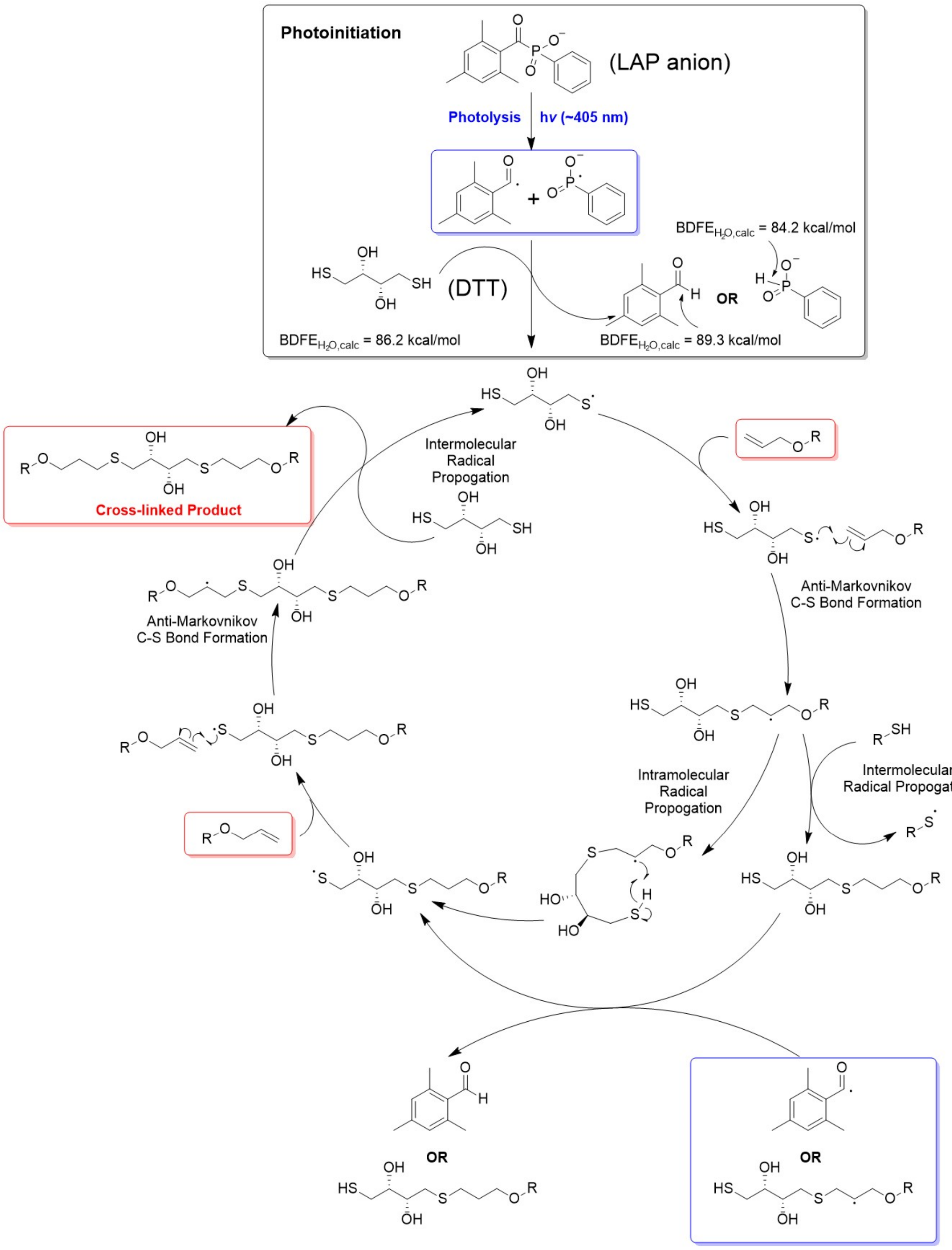

Figure S1. Mechanism of thiol-ene radical coupling mechanism, as employed in the reaction of allylated hydrogel precursors (GelAGE) with dithiothreitol (DTT) crosslinker and lithium phenyl-2,4,6-trimethylbenzoylphosphinate (LAP) photoinitiator.

The general mechanism of photoinitiated sulfur-ene radical coupling is well established [1], having first been reported by Kharasch, May, and Mayo in 1938 [2], and the mechanism remains accepted today [3]. A reasonable mechanism specific for the cross-coupling of GelAGE chains with DTT is presented in Figure S1. The coupling reaction is initiated by photolysis of the LAP anion with ~405 nm light generating a 2,4,6-trimethylbenzoyl radical and a phenylphosphonate radical anion [4, 5]. Initiation of the thiol-ene reaction begins via homolytic cleavage of the thiol S-H bond. The products of hydrogen atom abstraction for the two products are 2,4,6-trimethylbenzaldehyde and phenylphosphonate anion. In comparison with other photoinitiation systems, e.g. $[Ru(bpy)_3]^{2+}$/persulfate [6], this pair of radicals are mild, competent for hydrogen atom transfer (HAT) from S-H moieties while presenting low cytotoxicity; though Nguyen and coworkers have reported cytotoxicity of LAP for some lithium-sensitive cell lines [7].

To better understand the 'mildness' of the photogenerated radicals, their bond dissociation enthalpies (BDE) and aqueous bond dissociation free energies ($BDFE_{(aq)}$) were calculated using an experimentally calibrated method (*see section S.2. for details*). To our knowledge, none of the relevant BDEs/BDFEs have been experimentally determined for the species specific to our system. Thus, we can only compare the computational results in the context of similar molecules. In practice the reaction occurs in aqueous solution and therefore aqueous BDFEs provide the most appropriate comparison; however, condensed-phase BDFEs are more sparsely reported than BDEs for which many experimental methods exist. Thus, comparison of computational results with known BDEs was done complimentarily with aqueous BDFEs when available.

The $BDE_{(S\text{-}H)}$ of DTT was calculated to be 87.8 kcal/mol, in excellent agreement with experimentally derived aliphatic thiol S-H BDEs (87.7 ± 0.6 kcal/mol) [8]. The $BDFE_{(SH,\ aq)}$ of DTT was calculated to be 86.2 kcal/mol which is slightly higher than experimental $BDFE_{(SH,\ aq)}$ of reported aliphatic thiols (83.4 ± 1.0 kcal/mol) [9]. This elevated value can be rationalized by a relatively enhanced stabilization of the more polar dithiol/diol DTT reactant in water compared to reported aliphatic thiols [10]. We note here that the experimental $BDFE_{(SH,aq)}$ of cysteine is 81.6 kcal/mol [9], thus some degree of crosslinking of GelAGE is possible even in the absence of DTT. This was observed through increased viscosity of an experimental control, although the sample did not sufficiently solidify for mechanical characterization.

In order to provide the thermodynamic driving force for the initiation of the proposed self-propagating radical coupling mechanism, the conjugate hydrogen abstraction products of the LAP photolysis radicals should have a $BDFE_{(XH,aq)}$ greater than 86.2 kcal/mol. Neither product's relevant BDE/$BDFE_{(XH,aq)}$ are known, but the BDE of benzaldehyde (87.9 ± 2.77 kcal/mol) [8] and the BDEs of several mono-substituted benzaldehydes have been reported (82.9 ± 1.2 kcal/mol) [11]. The BDE of 2,4,6-trimethylbenzaldehyde was calculated to be 90.3 kcal/mol, in good agreement with reported unsubstituted benzaldehyde; for comparison, the methyl C-H BDE of toluene is not appreciably altered with mono- or di-methyl substitution at any ring position (the standard deviation is 0.3 kcal/mol for all reported species) [12]. This value is higher than the BDE of DTT, but lower than reported theoretical BDEs of amino acids, with the exception of cysteine SH, proline NH, tryptophan NH, and tyrosine OH [13]. The $BDFE_{(CH,aq)}$ of 2,4,6-trimethylbenzaldehyde was calculated to be 89.3 kcal/mol, greater than the calculated $BDFE_{(SH,aq)}$ of DTT, and therefore thermodynamically favorable. On the other hand, the phenylphosphonate $BDFE_{(PH,aq)}$ was calculated to be 84.2 kcal/mol, and therefore not competent for HAT from DTT. Unfortunately, while multiple reports agree that the 2,4,6-trimethylbenzoyl radical is the primary initiator, no reasonable comparison molecules for the phenylphosphonate radical anion have reported BDEs or BDFEs.

The actual thiyl-ene coupling reaction is an anti-Markovnikov C-S bond formation between the thiyl radical species and the terminal alkene moiety of the GelAGE precursor. Selectivity is governed by the significantly enhanced stability of the radical at the secondary position relative to a primary carbon radical. The driving force of this coupling reaction is estimated to be ~10 kcal/mol downhill, generating a potent secondary alkyl radical with estimated BDE ~95-99 kcal/mol [8]. This potent hydrogen abstractor carbon radical can then propagate the radical reaction through three reasonable pathways: 1) intramolecular radical propagation by HAT from the second DTT thiol S-H, 2) intermolecular radical propagation by HAT from an exogenous DTT thiol S-H, and 3) chain propagation through C-C alkyl radical/alkene coupling. Chain propagation has been reported to be kinetically disfavored by up to 3 orders of magnitude relative to propagation by HAT from thiols, and therefore likely contributes minimally (<1%) to cross-linking in our GelAGE systems; as such, we chose to omit it from Figure S1 [14].

Intramolecular radical propagation is feasible due to the 6-membered chain of DTT, supporting the formation of a 9-membered ring in the transition state of the putative hydrogen atom abstraction step. The produced thiyl radical can then couple with a second alkene to generate a cross-linked radical, followed by intermolecular radical propagation to generate the cross-linked product. On the other hand, intermolecular radical propagation must be followed by hydrogen atom abstraction from the second DTT thiol S-H; which can be accomplished by an additional 2,4,6-trimethylbenzoyl radical, or an exogenous alkyl radical. Subsequent steps are then identical to the intramolecular case, forming the cross-linked product and a DTT radical for further cross-linking at another site.

Radical termination is the final step of the process. The fate of the phenylphosphonate radical in LAP photoinitiated systems has been explicitly defined via ^{31}P NMR spectroscopy [15], with the primary products reported to be a hypophosphinate dimer and a phosphonothioic acid ester. Generally, termination of thiyl radicals occurs via the formation of disulfide bonds, which would be a complicating side-reaction in the selective crosslinking. However, cleavage of the disulfide bonds in thiol-ene click systems is known to be facile, indeed both thiyl radicals and even the weak phenylphosphonate radical have been shown to cleave disulfide bonds, further favoring the desired C-S crosslinking moiety [15, 16]. A similar rationale can be used to presume the possibility of disulfidation where a C-S bond is formed between thiyl/carbon radicals, irreversibly leading to potential intrapeptide linkages; however, recent reports suggest such linkage is kinetically prohibited with HAT from thiol proceeding an average of 30 times faster than thiyl/carbon radical coupling [14].

S.2. Computational Methods

S.2.1 General

Density functional theory (DFT) calculations were performed with the electronic structure package Orca Revision 5.0.3 [17]. Initial structures were built using relevant fragment bond lengths and angles as implemented in Chemcraft Version 1.8 [18]. All structures were optimized using the hybrid functional B3LYP [19-22] within the unrestricted Kohn-Sham formalism. The basis sets on O, P, and S atoms were of triple-ζ quality (Def2-TZVP), and double-ζ quality (Def2-SVP) basis sets on C and H atoms [23]. Models were optimized using default convergence criteria and integration grids as implemented in the Orca software package. To ensure stationary points on the potential energy surfaces were obtained and to provide zero-point energies, analytical frequency calculations were performed, and no imaginary frequencies were found. Solvated single-point energies were determined using the TPSSh functional [24], with the same basis sets as for geometry

optimizations, and the conductor-like polarized continuum (CPCM) [25] solvation model with the solvent parameters of water.

S.2.2. Determination of BDFE

The X-H BDEs and BDFEs were determined isodesmically against the reported experimental BDE/BDFE for the O-H bond of PhOH. Specifically, the calculated BDE and BDFEs were determined via equations 2 and 4, respectively:

$$\mathbf{\Delta H_{R-H,calc}} = (\mathbf{\Delta H_{R\cdot}} + \mathbf{\Delta H_{PhOH}}) - (\mathbf{\Delta H_{RH}} + \mathbf{\Delta H_{PhO\cdot}}) \quad \text{(S1)}$$

$$\mathbf{BDE_{R-H,calc}} = \mathbf{\Delta H_{R-H,calc}} + \mathbf{BDE_{PhOH}} \quad \text{(S2)}$$

$$\mathbf{\Delta G(R\text{-}H)_{calc,aq}} = (\mathbf{\Delta G_{R\cdot}} + \mathbf{\Delta G_{PhOH}}) - (\mathbf{\Delta G_{RH}} + \mathbf{\Delta G_{PhOH}}) \quad \text{(S3)}$$

$$\mathbf{BDFE(R\text{-}H)_{calc,aq}} = \mathbf{\Delta G(R\text{-}H)_{calc,aq}} + \mathbf{BDFE_{PhOH,aq}} \quad \text{(S4)}$$

where BDE_{PhOH} and $BDFE_{PhOH,aq}$ have the reported values 87.7 kcal/mol and 84.7 kcal/mol, respectively [9, 10]. For completeness, computational results are provided in Table S1. This mixed-functional/mixed-basis method has previously been shown to yield good accuracy in comparison with experimental BDEs/BDFEs [26, 27].

Table S1. Selected DFT-derived (Orca, B3LYP/TPSSh, Def2-TZVP{O,P,S}/Def2-SVP{C,H}) thermodynamic values discussed in this manuscript. Gas phase enthalpy ($H_{(B3LYP,gas)}$), gas phase Gibbs free energy ($G_{(B3LYP,gas)}$), gas phase single-point (SP(TPSSh,gas)), aqueous single-point (SP(TPSSh,aq)), and solvated Gibbs free energy (G(total,aq) are reported in Hartrees (Eh). Reaction thermodynamic energies and final BDE/BDFE values are reported in kcal/mol after multiplying resultant Eh energies by 627.5096 kcal mol^{-1} Eh^{-1}.

Species	$H_{(B3LYP,gas)}$(Eh)	$G_{(B3LYP,gas)}$(Eh)	$SP_{(TPSSh,gas)}$(Eh)	$SP_{(TPSSh,aq)}$(Eh)	$G_{(total,aq)}$(Eh)
PhO*	-306.4186163	-306.4536882	-306.7181076	-306.7277841	-306.4633647
PhOH	-307.0450927	-307.0805169	-307.3599136	-307.3692965	-307.0898998
$ArPO_2$*	-723.0939078	-723.1361528	-723.4904637	-723.5867486	-723.2324377
$ArPO_2H$	-723.7175612	-723.7591937	-724.127325	-724.226259	-723.8581277
DTT*	-1104.193204	-1104.240402	-1104.649137	-1104.663362	-1104.254627
DTT-H	-1104.819869	-1104.867777	-1105.289634	-1105.305471	-1104.883613
Me_3PhCO*	-462.1479086	-462.1957983	-462.6678505	-462.674782	-462.2027298
Me_3PhCO-H	-462.7785704	-462.8279278	-463.3146911	-463.3233877	-462.8366244

Reaction	ΔH(kcal/mol)	$\Delta G_{(total,H2O)}$(kcal/mol)
$ArPO_2H$ + PhO* → $ArPO_2$* + PhOH	-1.771497251	-0.530332915
DTT-H + PhO* → DTTrad + PhOH	0.118103582	1.53815958
Me_3PhCOH + PhO* → Me_3PhCO* + PhOH	2.626422606	4.618081119

BDE/BDFE	BDE (kcal/mol)	BDFE (kcal/mol)
PhOH → H* + PhO*	87.7	84.7
$ArPO_2H$ → H* + $ArPO_2$*	85.92850275	84.16966709
DTT-H → H* + DTT*	87.81810358	86.23815958
Me_3PhCOH → H* + Me_3PhCO*	90.32642261	89.31808112

S.3. Nuclear Magnetic Resonance (NMR) Spectroscopy

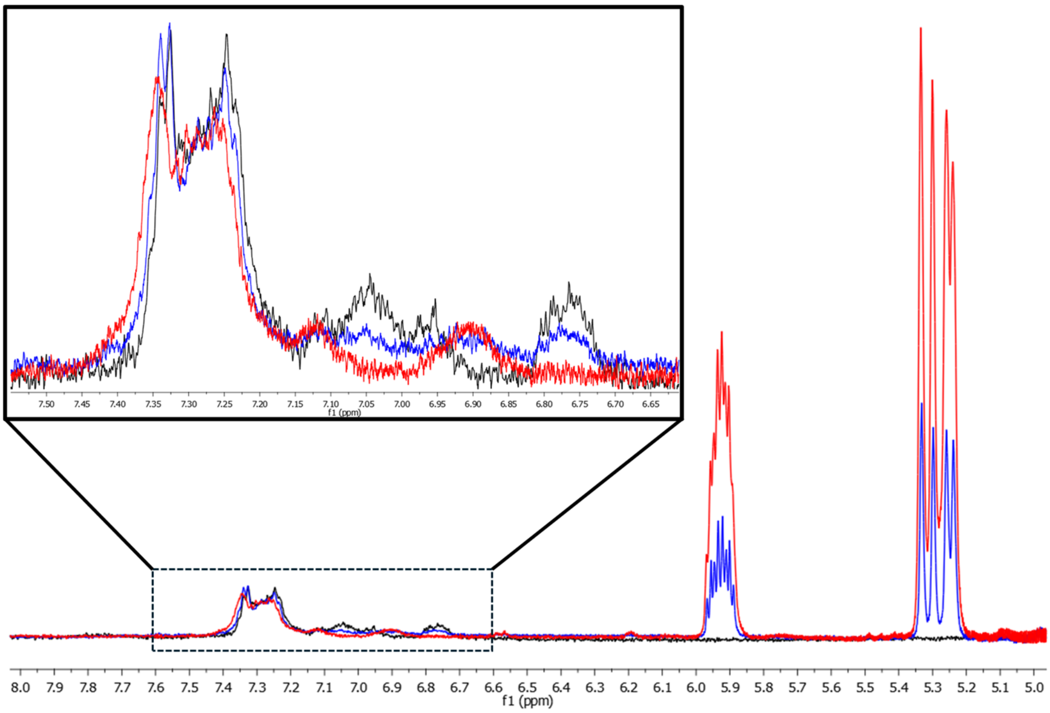

Figure S2. Superimposed ^{1}H-NMR spectrum including the aromatic and vinylic regions (δ = 5.0 – 8.0 ppm) of GelAGE-8MH (red), GelAGE-8LM (blue), and gelatin standard (black). The aromatic region (δ = 6.7-7.4 ppm) contains resonances diagnostic of phenylalanine (PHE-δ,ε,ζ, 5H), arginine (ARG-ε), asparagine (ASN-δ), aspartic acid (ASP-δ), glycine (GLN-ε), lysine (LYS-ζ), and possibly histidine (HIS-δ), and tyrosine (TYR-δ) [28]. Spectra were normalized to the aromatic region (δ = 7.15 – 7.45 ppm). Complex splitting patterns associated with the vinylic proton (δ = 5.86 – 5.98 ppm, quartet of triplets) and geminal protons (δ =5.22 – 5.35 ppm, doublet of doublets) highlight increasing relative degree of functionalization, with a ratio of 3.26:1 for 8MH:8LM (see Figure 5). Inset: The resonance tentatively assigned to ARG-ε (δ = 7.33 ppm) in the gelatin standard is retained in GelAGE-8LM, but is absent in GelAGE-8MH, consistent with functionalization of the ARG residue. Peaks tentatively assigned to ARG-η protons at δ = 6.77 and 7.04 ppm in the gelatin standard are present but broadened in GelAGE-8LM and apparently shift to δ = 6.91 and 7.13 ppm, respectively, in GelAGE-8MH.

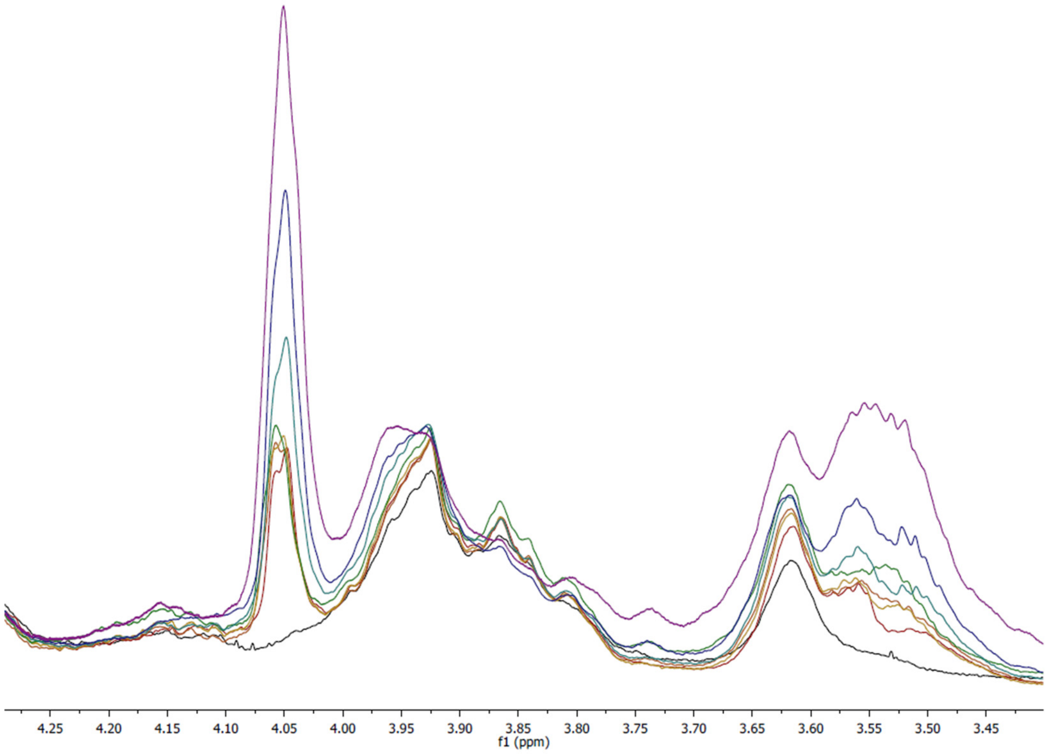

Figure S3. Superimposed ^{1}H-NMR spectra, normalized to the aromatic region from 7.2-7.4 ppm, showing evolution of peaks consistent with proton resonances diagnostic of incorporation of the AGE group. GelAGE samples 8LL, 4LM, 8LM, and 8LH (red, orange, brown, green, respectively) are nominally equivalent (<5% difference in peak integrations) the remaining three, 1MM (teal), 8MM (indigo), and 8MH (purple) show increasing functionalization.

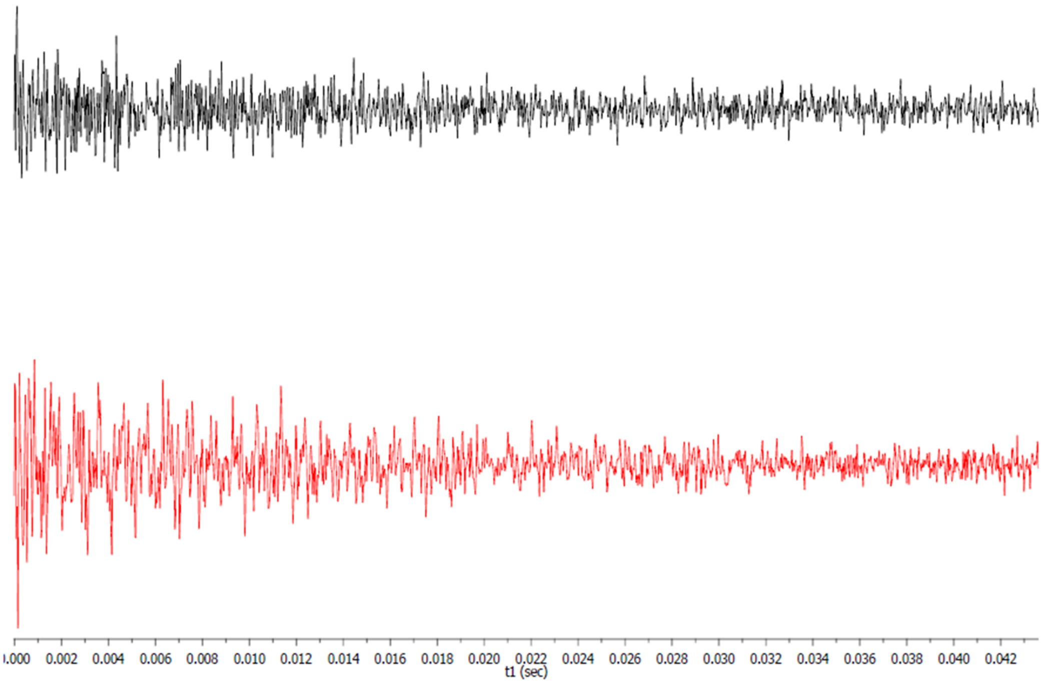

Figure S4. Representative raw ^{13}C-NMR free induction decay (FID) spectra of GelAGE-4LM (black) and GelAGE-8MH (red). T2* shortening attributable to field inhomogeneity due to molecular aggregation results in rapid loss of signal (< 20 ms) despite accumulation of 32,768 transients (~19 hour total collection time).

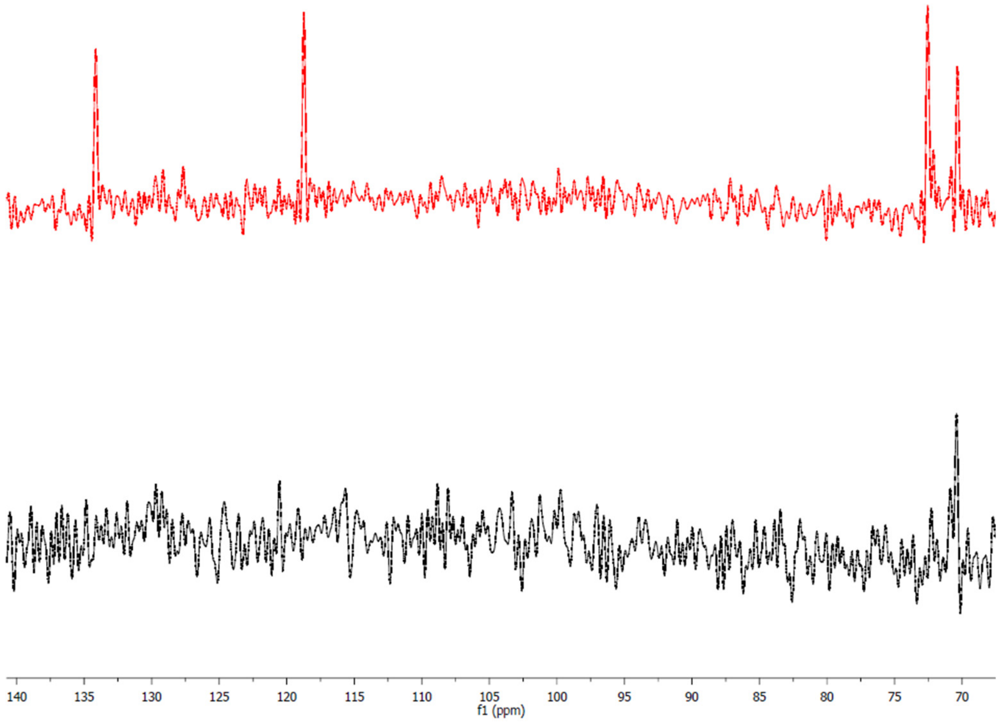

Figure S5. Representative ^{13}C-NMR of GelAGE-4LM (red) vs gelatin standard (black) showing emergence of peaks diagnostic of carbons bearing vinylic and geminal protons at 134.2 and 118.8 ppm, respectively, and a peak consistent with a carbon adjacent to an ether and alcohol at 72.6 ppm.

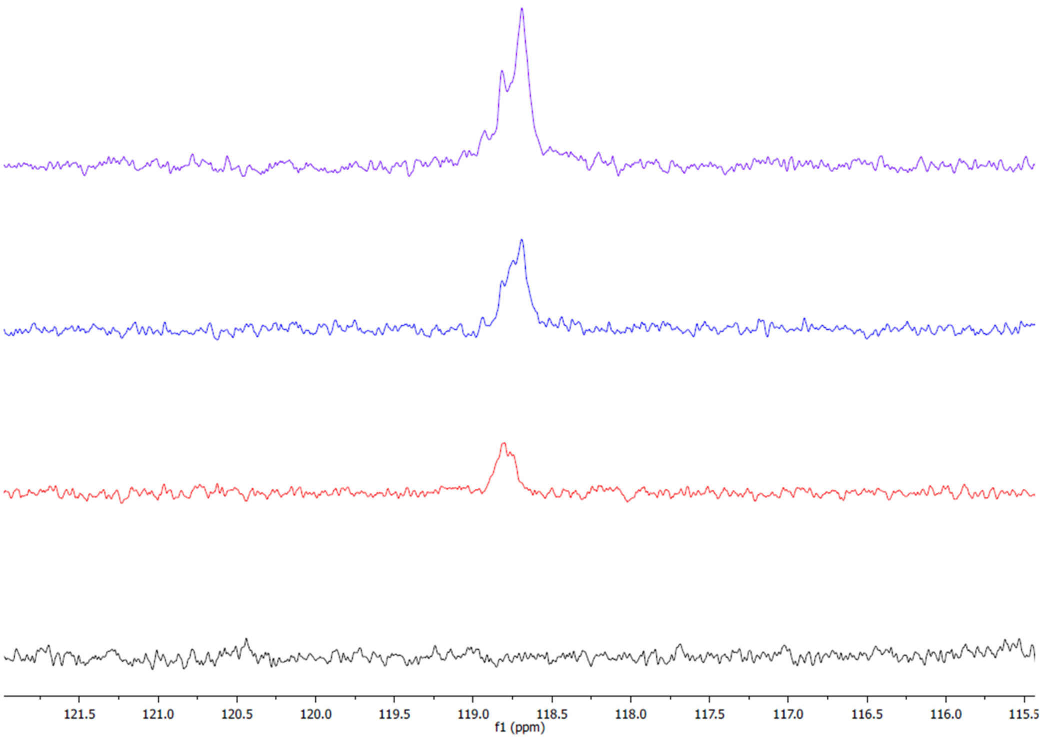

Figure S6. Stacked ^{13}C-NMR spectra of Gelatin Standard (Black), GelAGE-4LM (red), GelAGE-8MM (blue), and GelAGE-8MH (purple) showing evolution of peaks consistent with three unique carbons bearing geminal protons at 118.80, 118.75, and 118.70 ppm. All GelAGE spectra show the initial 118.80 ppm peak assigned to functionalized lysine residues. The two up-field shifted peaks observed in GelAGE-8MM are tentatively assigned to singly- and doubly-functionalized arginine residues. GelAGE-8MH shows increased concentration of the 118.70 ppm peak.

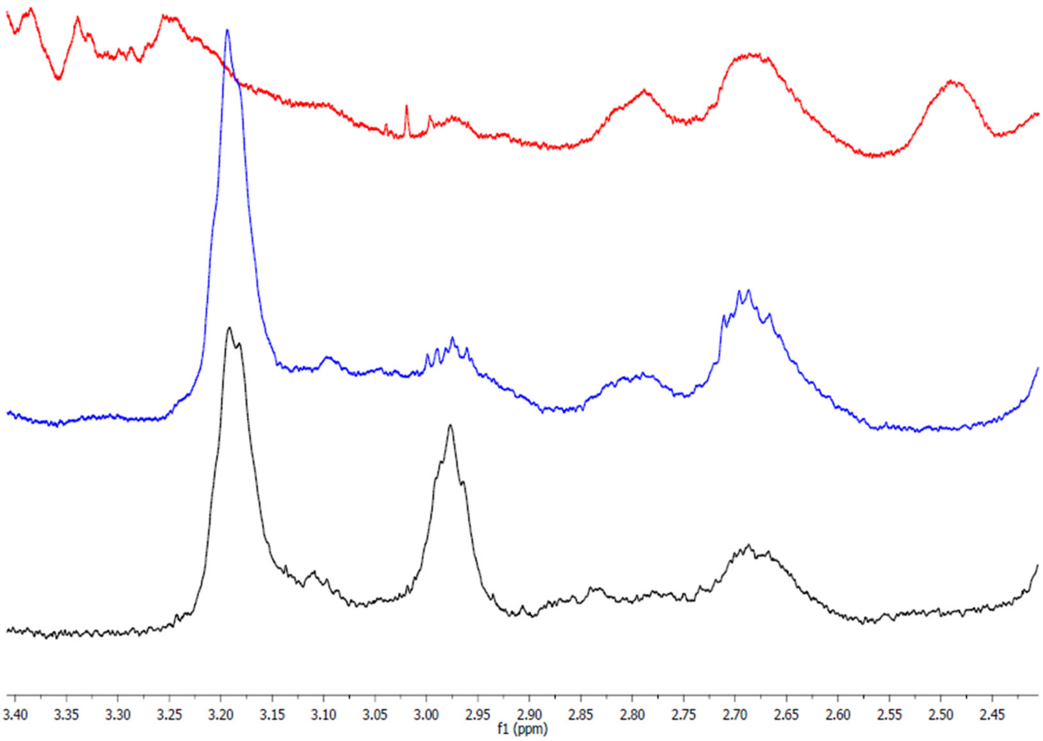

Figure S7. Stacked ^{1}H-NMR spectrum of key region (δ = 2.3 – 3.4 ppm) containing diagnostic amino acid proton resonance changes upon functionalization with AGE for GelAGE-8MH (red), GelAGE-8LM (blue), and gelatin standard (black). The sharp feature observed at δ = 2.98 ppm is assigned by 1D-TOCSY (see Figure S8) primarily to the LYS-ε proton resonances. Its attenuation coincides with the appearance of a broad feature at δ = 2.80 ppm as observed in both the GelAGE-8LM and GelAGE-8MH samples. The sharp feature at δ = 3.19 ppm is assigned by 1D-TOCSY (see Figure S9) primarily to the ARG-δ proton resonances and is retained in GelAGE8LM; its attenuation in GelAGE-8MH coincides with the appearance of a broad feature at δ = 2.48 ppm, assigned by 1D-TOCSY (see Figure S10) as AGE-functionalized-ARG-γ (*f*ARG-γ) proton resonances. Other unassigned features are observed at δ = 3.24 – 3.39 ppm, on the tail of the intense AGE band observed at δ = 3.55 ppm.

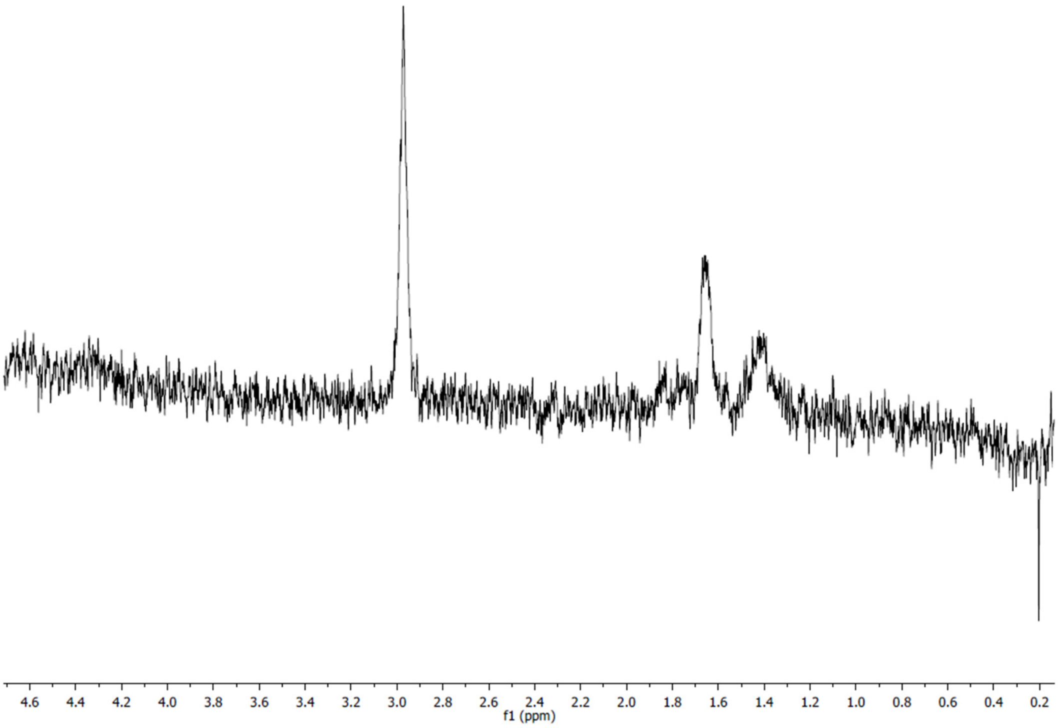

Figure S8. Selective 1D-TOCSY (2.98 ppm, Δ = 36 Hz, 80 ms mix-time) of Gelatin Standard. Strong correlation observed at 1.65 ppm, and moderate correlation observed at 1.42 ppm, consistent with assignments as LYS-δ and LYS-γ protons, respectively. These correlations unambiguously assign the 2.98 ppm resonance to LYS-ε protons.

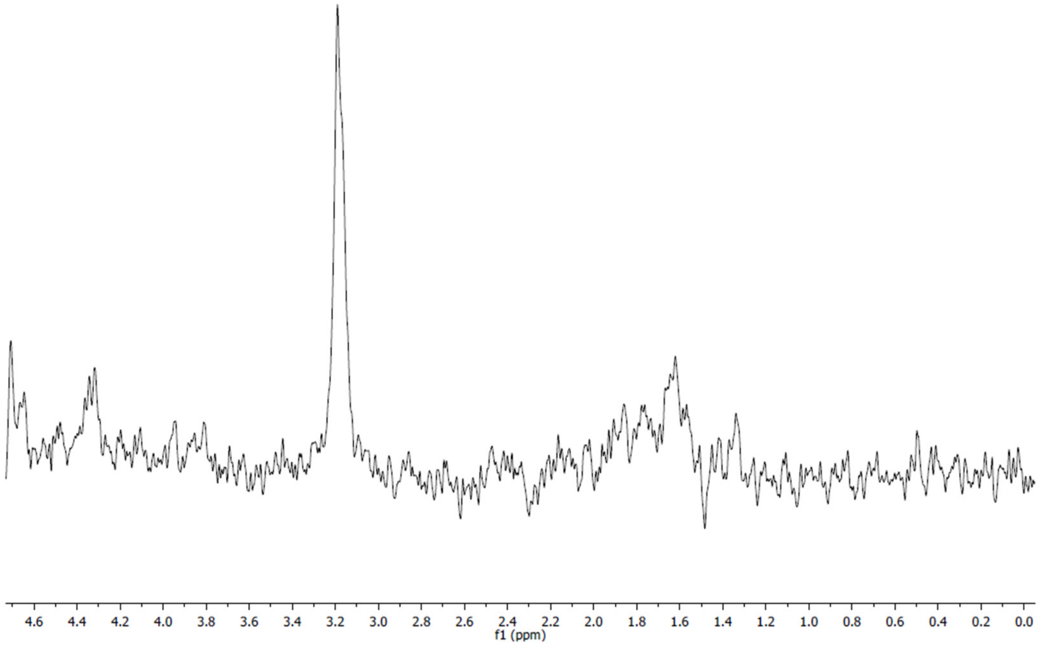

Figure S9. Selective 1D-TOCSY (3.19 ppm, Δ = 36 Hz, 80 ms mix-time) of Gelatin Standard. Moderate correlation with peaks at 1.63 ppm and weaker correlation with a peak at 1.86 ppm are consistent with assignment as ARG-γ and ARG-β protons, respectively. These correlations tentatively assign the 3.19 ppm feature as ARG-δ protons.

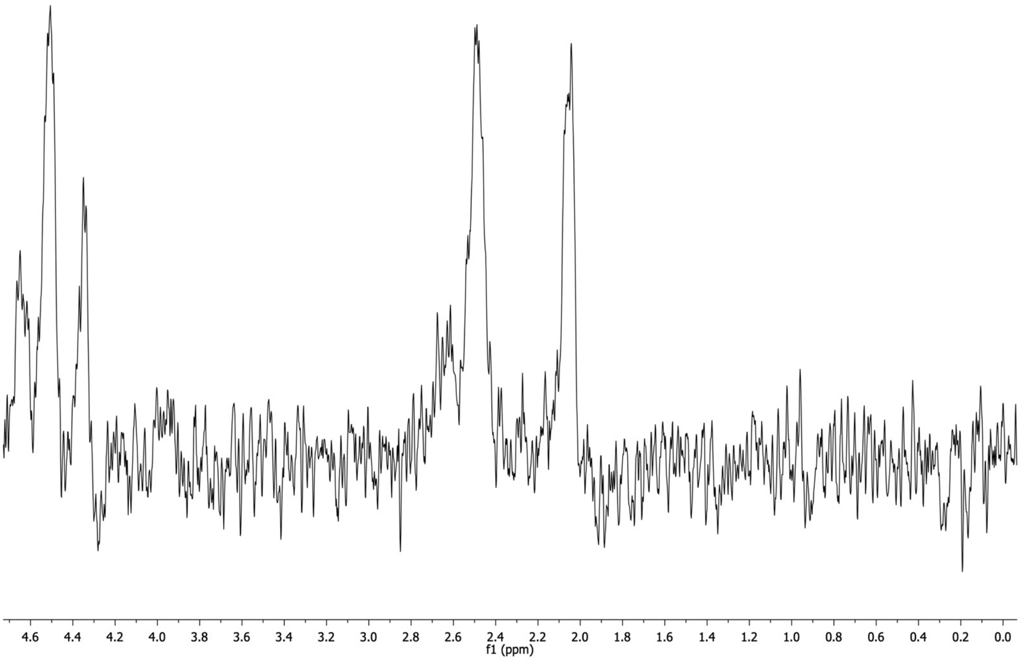

Figure S10. Selective 1D-TOCSY (2.48 ppm, Δ = 36 Hz, 80 ms mix-time) of GelAGE-8MH. Strong correlation with peaks at 4.52 and 2.05 ppm, and weaker correlation with peaks at 4.35 ppm allow assignment of each as *f*ARG-δ, *f*ARG-β, and *f*ARG-α protons respectively. Consistent with assignment of the 2.48 ppm feature to *f*ARG-γ protons.

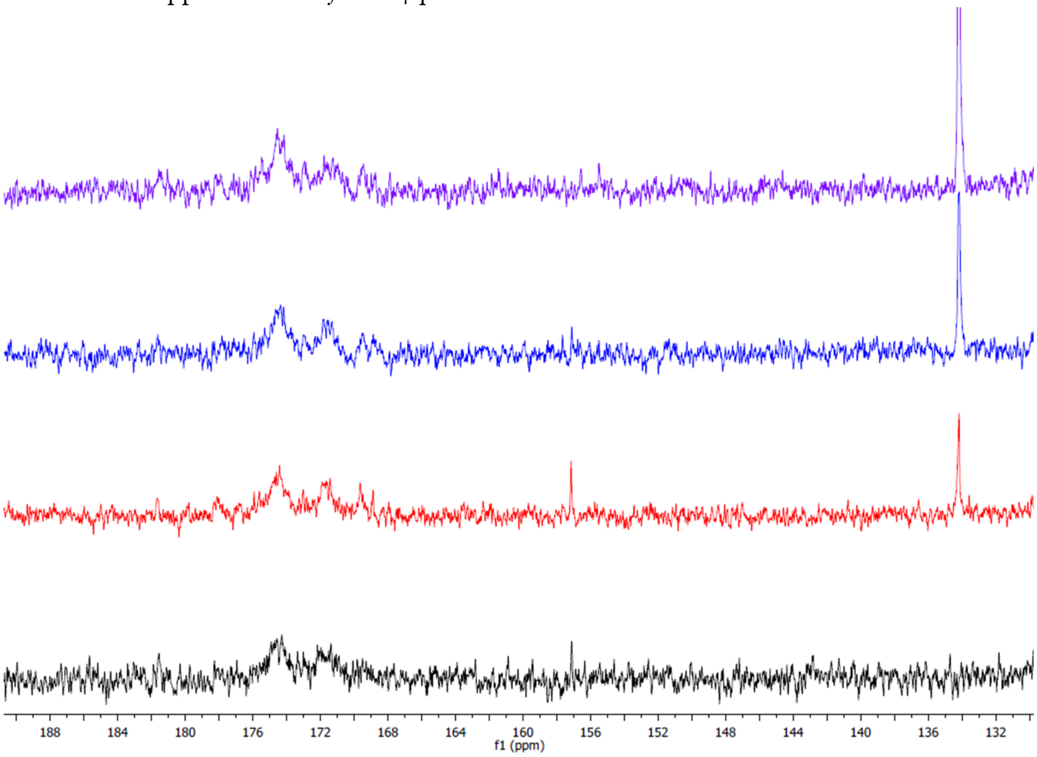

Figure S11. ^{13}C-NMR spectra of Gelatin Standard (black), GelAGE-4LM (red), GelAGE-8MM (blue), and GelAGE-8MH (purple) showing attenuation of the diagnostic ARG-ζ carbon resonance at 157.1 ppm as a function of increasing arginine functionalization across the series.

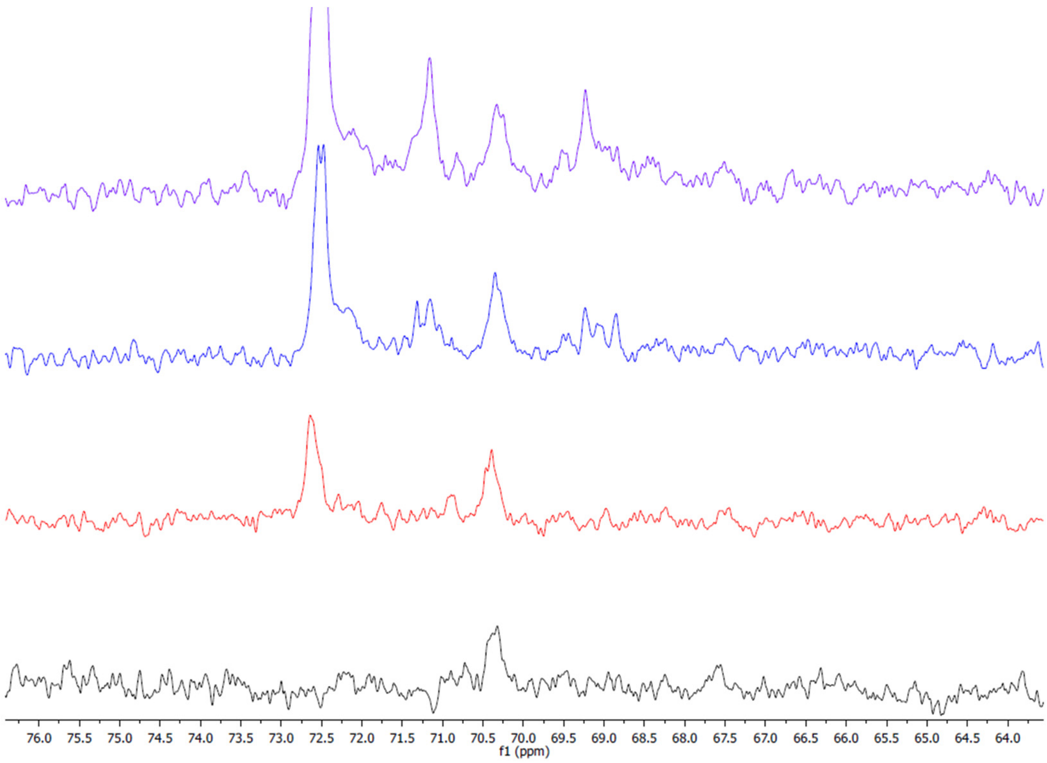

Figure S12. ^{13}C-NMR spectra of Gelatin Standard (black), GelAGE-4LM (red), GelAGE-8MM (blue), and GelAGE-8MH (purple) showing evolution of multiple resonances at ca. 69.1 and 71.2 ppm in GelAGE-8MM, which coalesce into two features in GelAGE-8MH with increasing functionalization.

Amino Acid Residue Chemical Shift Comparison Gelatin Standard vs GelAGE					
**Residue Proton**	**BMRB Average[a]**	**Gel. Std.**	**Funct. Residue Proton**	**GelAGE**	**Difference[b]**
LYS-α	4.26	--			
LYS-β	1.77	--			
LYS-γ	1.36	1.43[c]			
LYS-δ	1.61	1.66[c]			
LYS-ε	2.91	2.98[c]			
ARG-α	4.29	4.45[c]	*f*ARG-α	4.52[c]	0.07
ARG-β	1.79	1.86[c]	*f*ARG-β	2.48[c]	0.62
ARG-γ	1.56	1.63[c]	*f*ARG-γ	2.05[c]	0.42
ARG-δ	3.11	3.19[c]	*f*ARG-δ	4.35[c]	1.16
ARG-ε	7.35	7.33[d]	*f*ARG-ε	7.37[d]	0.04
ARG-η_1	6.87	7.04[d]	*f*ARG-η_1	7.13[d]	0.09
ARG-η_2	6.79	6.77[d]	*f*ARG-η_2	6.91[d]	0.14

a: Average residue proton chemical shift value reported by the Biological Magnetic Resonance Data Bank [28].

b: Difference relative to value observed of analogous unfunctionalized residue in gelatin standard.

c: Value derived from selective 1D-TOCSY data

d: Tentative value derived from inspection of ^{1}H-NMR (see Figure S2)

Table S2. Comparison table of average residue proton positions reported in the Biological Magnetic Resonance Data Bank (BMRB) [28] vs. values obtained from NMR spectroscopy reported in this work. All values obtained for residues in the gelatin standard lie within the standard deviation values reported by BMRB for the respective resonances.

S.4. Compressive Modulus

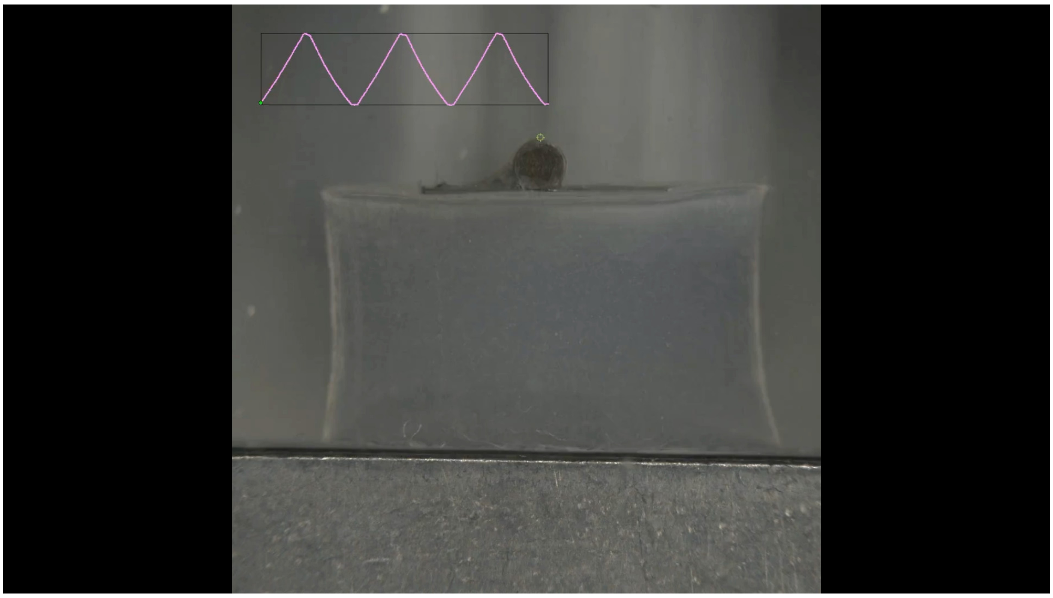

Video S1. In-instrument view of cyclic compression testing (100s cycle) of cross-linked GelAGE 8MH (CellScale, Waterloo, ON, Canada). At the top left, the green tracking dot represents the current position along the Force vs. Time graph. https://youtu.be/gp0m0c_BuFQ

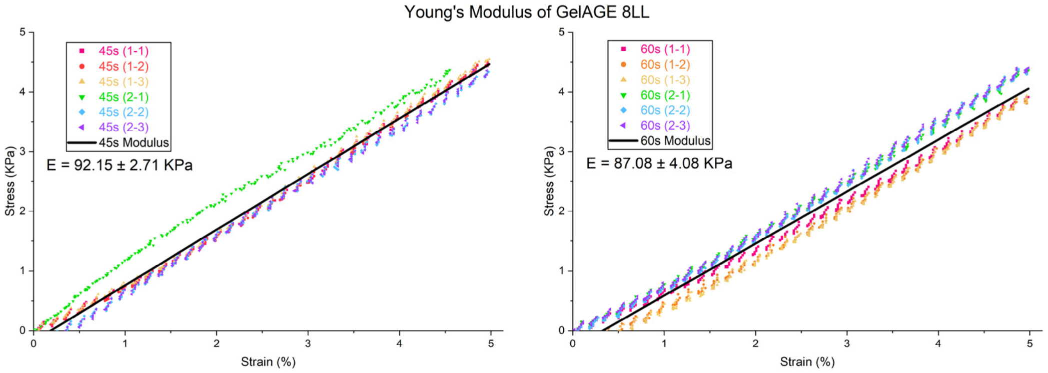

Figure S13. Compressive moduli of saturated GelAGE-8LL hydrogel, collected under physiological conditions (aqueous, 37 °C) via MicroTester (CellScale) in 100 s cycle (45 s compression) and 140 s cycle (60 s compression).

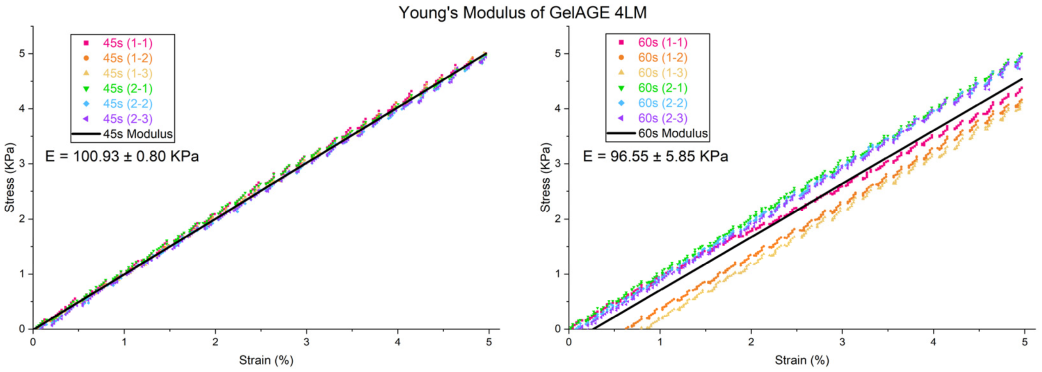

Figure S14. Compressive moduli of saturated GelAGE-4LM hydrogel, collected under physiological conditions (aqueous, 37 °C) via MicroTester (CellScale) in 100 s cycle (45 s compression) and 140 s cycle (60 s compression).

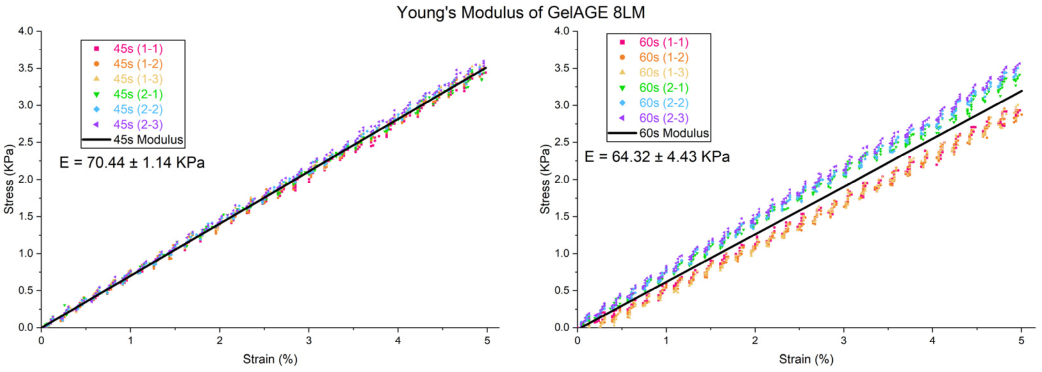

Figure S15. Compressive moduli of saturated GelAGE-8LM hydrogel, collected under physiological conditions (aqueous, 37 °C) via MicroTester (CellScale) in 100 s cycle (45 s compression) and 140 s cycle (60 s compression).

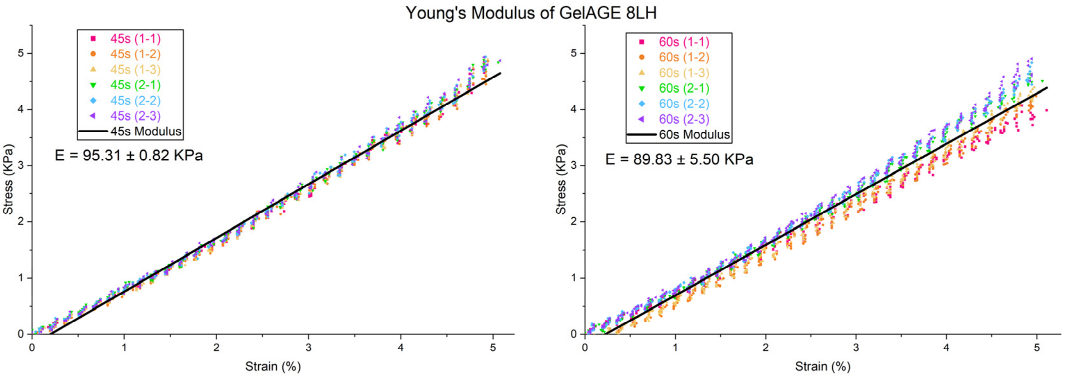

Figure S16. Compressive moduli of saturated GelAGE-8LH hydrogel, collected under physiological conditions (aqueous, 37 °C) via MicroTester (CellScale) in 100 s cycle (45 s compression) and 140 s cycle (60 s compression).

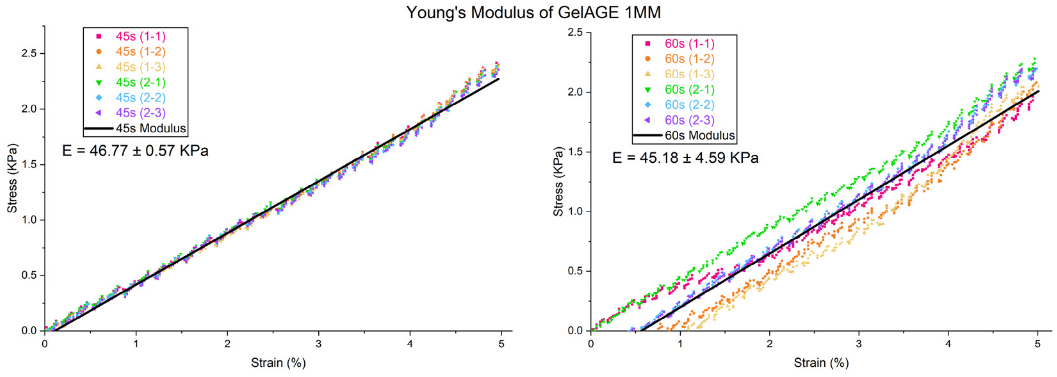

Figure S17. Compressive moduli of saturated GelAGE-1MM hydrogel, collected under physiological conditions (aqueous, 37 °C) via MicroTester (CellScale) in 100 s cycle (45 s compression) and 140 s cycle (60 s compression).

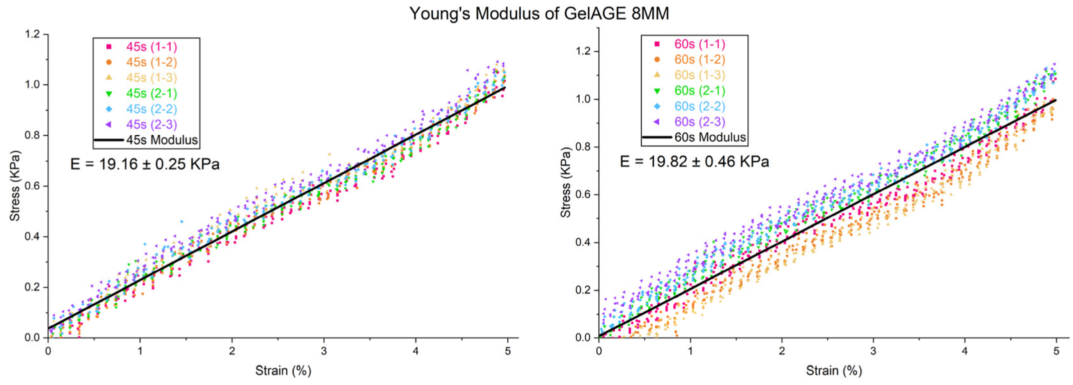

Figure S18. Compressive moduli of saturated GelAGE-8MM hydrogel, collected under physiological conditions (aqueous, 37 °C) via MicroTester (CellScale) in 100 s cycle (45 s compression) and 140 s cycle (60 s compression).

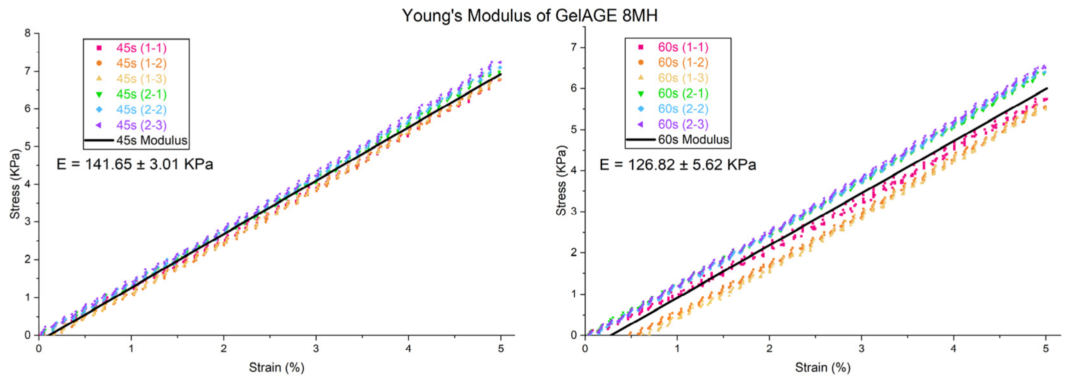

Figure S19. Compressive moduli of saturated GelAGE-8MH hydrogel, collected under physiological conditions (aqueous, 37 °C) via MicroTester (CellScale) in 100 s cycle (45 s compression) and 140 s cycle (60 s compression).

S.5. Degree of Swelling

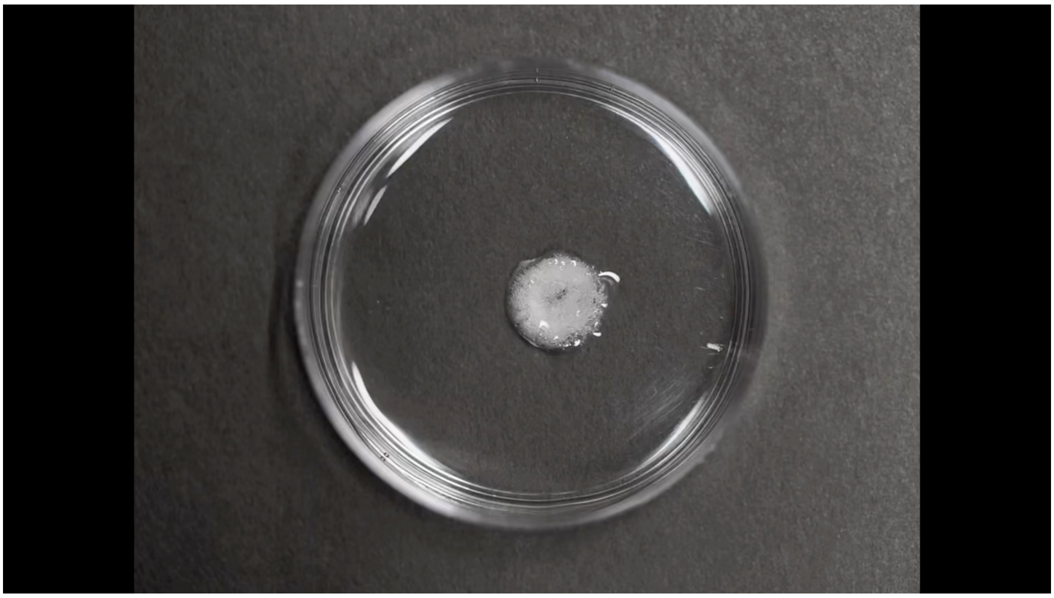

Video S2. 6 hour time-lapse (Olympus OM-1 equipped with Olympus M. Zuiko Digital ED 60mm f2.8 Macro Lens, OM Digital Solutions, Bethlehem, PA, USA) depicting the swelling behavior of lyophilized GelAGE (4LM) hydrogel. The petri dish (FisherBrand, Thermo Fisher Scientific, Waltham, MA, USA) measures 35 mm in diameter. https://youtu.be/w-gfS24I9BE

**Map Sum Spectrum**				
**Element**	**Line Type**	**Weight %**	**Weight % Sigma**	**Atomic %**
C	K series	53.91	0.32	60.86
N	K series	19.57	0.39	18.94
O	K series	20.67	0.17	17.52
S	K series	0.23	0.01	0.10
P	K series	0.08	0.01	0.04
Na	K series	1.28	0.02	0.75
K	K series	0.03	0.01	0.01
Cl	K series	2.86	0.03	1.09
Al	K series	1.37	0.02	0.69
Total		100.00		100.00

Table S3. EDX map sum spectra of gelatin (type A) standard, physically cross-linked at room temperature and lyophilized for SEM analysis and elemental detection (Oxford Instruments). Sulfur is highlighted in pink, in accordance with color grading on the EDX map (Figure 12).

Map Sum Spectrum				
**Element**	**Line Type**	**Weight %**	**Weight % Sigma**	**Atomic %**
C	K series	54.06	0.57	60.95
N	K series	18.65	0.70	18.03
O	K series	21.90	0.32	18.54
S	K series	1.12	0.03	0.47
P	K series	0.20	0.02	0.09
Na	K series	1.66	0.04	0.98
K	K series	0.03	0.02	0.01
Cl	K series	2.22	0.04	0.85
Al	K series	0.17	0.02	0.08
Total		100.00		100.00

Table S4. EDX map sum spectra of GelAGE-4LM, chemically cross-linked with DTT and LAP under UV irradiation for 2 minutes, and lyophilized for SEM analysis and elemental detection (Oxford Instruments). Sulfur is highlighted in pink, in accordance with color grading on the EDX map (Figure 12).

References

[1] A. B. Lowe, "Thiol-ene “click” reactions and recent applications in polymer and materials synthesis," *Polym. Chem.,* vol. 1, *(1),* pp. 17–36, 2010. Available: http://dx.doi.org/10.1039/B9PY00216B.

[2] T. Posner, "Beiträge zur Kenntniss der ungesättigten Verbindungen. II. Ueber die Addition von Mercaptanen an ungesättigte Kohlenwasserstoffe," *Ber. Dtsch. Chem. Ges.,* vol. 38, *(1),* pp. 646–657, 1905. Available: https://doi.org/10.1002/cber.190503801106.

[3] N. B. Cramer and C. N. Bowman, "Kinetics of thiol–ene and thiol–acrylate photopolymerizations with real-time fourier transform infrared," *J. Polym. Sci. A Polym. Chem.,* vol. 39, *(19),* pp. 3311–3319, 2001. Available: https://doi.org/10.1002/pola.1314.

[4] T. Majima, W. Schnabel and W. Weber, "Phenyl-2,4,6-trimethylbenzoylphosphinates as water-soluble photoinitiators. Generation and reactivity of O Ṗ(C6H5)(O−) radical anions," *Makromol. Chem.,* vol. 192, *(10),* pp. 2307–2315, 1991. Available: https://doi.org/10.1002/macp.1991.021921010.

[5] B. D. Fairbanks, M. P. Schwartz, C. N. Bowman and K. S. Anseth, "Photoinitiated polymerization of PEG-diacrylate with lithium phenyl-2,4,6-trimethylbenzoylphosphinate: polymerization rate and cytocompatibility," *Biomaterials,* vol. 30, *(35),* pp. 6702–6707, 2009. Available: https://www.sciencedirect.com/science/article/pii/S0142961209009041.

[6] D. A. Fancy and T. Kodadek, "Chemistry for the analysis of protein–protein interactions: Rapid and efficient cross-linking triggered by long wavelength light," *Proceedings of the National Academy of Sciences,* vol. 96, *(11),* pp. 6020–6024, 1999. Available: https://doi.org/10.1073/pnas.96.11.6020.

[7] A. K. Nguyen, P. L. Goering, R. K. Elespuru, S. Sarkar Das and R. J. Narayan, "The Photoinitiator Lithium Phenyl (2,4,6-Trimethylbenzoyl) Phosphinate with Exposure to 405 nm Light Is Cytotoxic to Mammalian Cells but Not Mutagenic in Bacterial Reverse Mutation Assays," *Polymers,* vol. 12, *(7),* 2020. .

[8] Y. Luo, "Comprehensive handbook of chemical bond energies," in 2007, Available: https://api.semanticscholar.org/CorpusID:92942042.

[9] R. G. Agarwal, S. C. Coste, B. D. Groff, A. M. Heuer, H. Noh, G. A. Parada, C. F. Wise, E. M. Nichols, J. J. Warren and J. M. Mayer, "Free Energies of Proton-Coupled Electron Transfer Reagents and Their Applications," *Chem. Rev.,* vol. 122, *(1),* pp. 1–49, 2022. Available: https://doi.org/10.1021/acs.chemrev.1c00521.

[10] J. J. Warren, T. A. Tronic and J. M. Mayer, "Thermochemistry of Proton-Coupled Electron Transfer Reagents and its Implications," *Chem. Rev.*, vol. 110, *(12)*, pp. 6961–7001, 2010. Available: https://doi.org/10.1021/cr100085k.

[11] V. E. Tumanov, E. A. Kromkin and E. T. Denisov, "Estimation of dissociation energies of C—H bonds in oxygen-containing compounds from kinetic data for radical abstraction reactions," *Russian Chemical Bulletin*, vol. 51, *(9)*, pp. 1641–1650, 2002. Available: https://doi.org/10.1023/A:1021335001753.

[12] E. T. Denisov and V. E. Tumanov, "Estimation of the bond dissociation energies from the kinetic characteristics of liquid-phase radical reactions," vol. 74, *(9)*, pp. 825, 2005. Available: https://doi.org/10.1070/RC2005v074n09ABEH001177.

[13] B. Chen, X. Ouyang, C. Cheng, D. Chen, J. Su, Y. Hu and X. Li, "Bioactive peptides derived from Radix Angelicae sinensis inhibit ferroptosis in HT22 cells through direct Keap1–Nrf2 PPI inhibition," *RSC Adv.*, vol. 13, *(32)*, pp. 22148–22157, 2023. Available: http://dx.doi.org/10.1039/D3RA04057G.

[14] S. M. Soars, N. J. Bongiardina, B. D. Fairbanks, M. Podgórski and C. N. Bowman, "Spatial and Temporal Control of Photomediated Disulfide–Ene and Thiol–Ene Chemistries for Two-Stage Polymerizations," *Macromolecules*, vol. 55, *(5)*, pp. 1811–1821, 2022. Available: https://doi.org/10.1021/acs.macromol.1c02464.

[15] B. D. Fairbanks, S. P. Singh, C. N. Bowman and K. S. Anseth, "Photodegradable, Photoadaptable Hydrogels via Radical-Mediated Disulfide Fragmentation Reaction," *Macromolecules*, vol. 44, *(8)*, pp. 2444–2450, 2011. Available: https://doi.org/10.1021/ma200202w.

[16] N. J. Bongiardina, S. M. Soars, M. Podgorski and C. N. Bowman, "Radical-disulfide exchange in thiol–ene–disulfidation polymerizations," *Polym. Chem.*, vol. 13, *(27)*, pp. 3991–4003, 2022. Available: http://dx.doi.org/10.1039/D2PY00172A.

[17] F. Neese, "Software update: The ORCA program system—Version 5.0," *WIREs Comput Mol Sci*, vol. 12, *(5)*, pp. e1606, 2022. Available: https://doi.org/10.1002/wcms.1606.

[18] Anonymous "Chemcraft - graphical software for visualization of quantum chemistry computations," vol. 1.8, .

[19] P. J. Stephens, F. J. Devlin, C. F. Chabalowski and M. J. Frisch, "Ab Initio Calculation of Vibrational Absorption and Circular Dichroism Spectra Using Density Functional Force Fields," *J. Phys. Chem.*, vol. 98, *(45)*, pp. 11623–11627, 1994. Available: https://doi.org/10.1021/j100096a001.

[20] S. H. Vosko, L. Wilk and M. Nusair, "Accurate spin-dependent electron liquid correlation energies for local spin density calculations: a critical analysis," *Can. J. Phys.*, vol. 58, *(8)*, pp. 1200–1211, 1980. Available: https://doi.org/10.1139/p80-159.

[21] C. Lee, W. Yang and R. G. Parr, "Development of the Colle-Salvetti correlation-energy formula into a functional of the electron density," vol. 37, *(2)*, pp. 785–789, 1988. Available: https://link.aps.org/doi/10.1103/PhysRevB.37.785.

[22] A. D. Becke, "Density-functional thermochemistry. III. The role of exact exchange," *J. Chem. Phys.*, vol. 98, *(7)*, pp. 5648–5652, 1993. Available: https://doi.org/10.1063/1.464913.

[23] F. Weigend and R. Ahlrichs, "Balanced basis sets of split valence, triple zeta valence and quadruple zeta valence quality for H to Rn: Design and assessment of accuracy," *Phys. Chem. Chem. Phys.*, vol. 7, *(18)*, pp. 3297–3305, 2005. Available: http://dx.doi.org/10.1039/B508541A.

[24] V. N. Staroverov, G. E. Scuseria, J. Tao and J. P. Perdew, "Comparative assessment of a new nonempirical density functional: Molecules and hydrogen-bonded complexes," *J. Chem. Phys.*, vol. 119, *(23)*, pp. 12129–12137, 2003. Available: https://doi.org/10.1063/1.1626543.

[25] M. Cossi, N. Rega, G. Scalmani and V. Barone, "Energies, structures, and electronic properties of molecules in solution with the C-PCM solvation model," *J. Comput. Chem.*, vol. 24, *(6)*, pp. 669–681, 2003. Available: https://doi.org/10.1002/jcc.10189.

[26] P. E. VanNatta, D. A. Ramirez, A. R. Velarde, G. Ali and M. Kieber-Emmons, "Exceptionally High O–H Bond Dissociation Free Energy of a Dicopper(II) μ-Hydroxo Complex and Insights into the Geometric and Electronic Structure Origins Thereof," *J. Am. Chem. Soc.*, vol. 142, *(38)*, pp. 16292–16312, 2020. Available: https://doi.org/10.1021/jacs.0c06425.

[27] M. Kieber-Emmons, J. W. Ginsbach, P. K. Wick, H. R. Lucas, M. E. Helton, B. Lucchese, M. Suzuki, A. D. Zuberbühler, K. D. Karlin and E. I. Solomon, "Observation of a CuII2(μ-1,2-peroxo)/CuIII2(μ-oxo)2 Equilibrium and its Implications for Copper–Dioxygen Reactivity," *Angew. Chem. Int. Ed.*, vol. 53, *(19)*, pp. 4935–4939, 2014. Available: https://doi.org/10.1002/anie.201402166.

[28] J. C. Hoch, K. Baskaran, H. Burr, J. Chin, H. R. Eghbalnia, T. Fujiwara, M. R. Gryk, T. Iwata, C. Kojima, G. Kurisu, D. Maziuk, Y. Miyanoiri, J. R. Wedell, C. Wilburn, H. Yao and M. Yokochi, "Biological Magnetic Resonance Data Bank," *Nucleic Acids Res.*, vol. 51, pp. D368–D376, 2023. Available: https://doi.org/10.1093/nar/gkac1050.
